# Chemical screening by time-resolved X-ray scattering to discover allosteric probes

**DOI:** 10.1038/s41589-024-01609-1

**Published:** 2024-04-26

**Authors:** Chris A. Brosey, Todd M. Link, Runze Shen, Davide Moiani, Kathryn Burnett, Greg L. Hura, Darin E. Jones, John A. Tainer

**Affiliations:** 1 Department of Molecular and Cellular Oncology, The University of Texas MD Anderson Cancer Center, Houston, TX USA; 2 MBIB Division, Lawrence Berkeley National Laboratory, Berkeley, CA USA; 3 Department of Chemistry and Biochemistry, University of California, Santa Cruz, Santa Cruz, CA USA; 4 Department of Pharmaceutical Sciences, University of Arkansas for Medical Sciences, Little Rock, AR USA; 5 Department of Cancer Biology, The University of Texas MD Anderson Cancer Center, Houston, TX USA

**Keywords:** Screening, Small molecules, Structural biology, Medicinal chemistry, Enzyme mechanisms

## Abstract

Drug discovery relies on efficient identification of small-molecule leads and their interactions with macromolecular targets. However, understanding how chemotypes impact mechanistically important conformational states often remains secondary among high-throughput discovery methods. Here, we present a conformational discovery pipeline integrating time-resolved, high-throughput small-angle X-ray scattering (TR-HT-SAXS) and classic fragment screening applied to allosteric states of the mitochondrial import oxidoreductase apoptosis-inducing factor (AIF). By monitoring oxidized and X-ray-reduced AIF states, TR-HT-SAXS leverages structure and kinetics to generate a multidimensional screening dataset that identifies fragment chemotypes allosterically stimulating AIF dimerization. Fragment-induced dimerization rates, quantified with time-resolved SAXS similarity analysis (*k*_VR_), capture structure–activity relationships (SAR) across the top-ranked 4-aminoquinoline chemotype. Crystallized AIF–aminoquinoline complexes validate TR-SAXS-guided SAR, supporting this conformational chemotype for optimization. AIF–aminoquinoline structures and mutational analysis reveal active site F482 as an underappreciated allosteric stabilizer of AIF dimerization. This conformational discovery pipeline illustrates TR-HT-SAXS as an effective technology for targeting chemical leads to important macromolecular states.

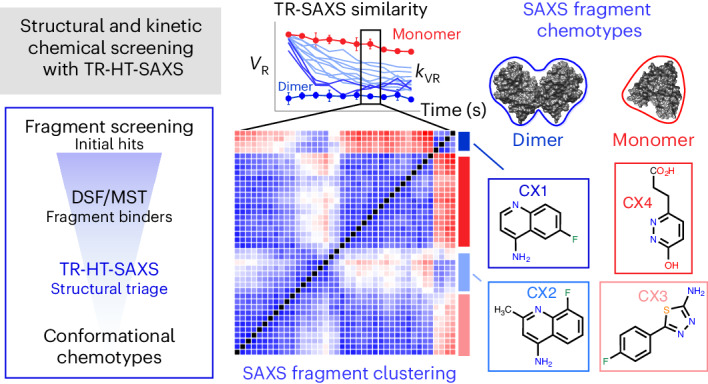

## Abstract (web summary)

A discovery pipeline integrating time-resolved HT-SAXS and fragment screening identifies chemical leads targeting exemplary allosteric states of mitochondrial oxidoreductase apoptosis-inducing factor (AIF).

## Main

Fragment-based drug discovery and optimization powerfully identify small-molecule leads for research and clinical applications by established technologies^1^. Yet, ligand binding effects on target structure and/or dynamics are typically only examined in later development phases. Biomolecular conformation drives function for many targets of high biological importance, including allosteric assemblies^2,3^, protein–protein interfaces^4^, intrinsically disordered proteins (IDPs)^5^ and nucleic acids^6^. Specific targeting of these mechanistically important structural states with chemical ligands provides an avenue for greater precision and potency in modulating their biological functions. Thus, early discovery of assembly- or conformation-specific chemical scaffolds is well positioned to accelerate ligand optimization to specific biomolecular functions. Additionally, structural biology’s increasing recognition of conformational ensembles with dynamic equilibria, such as IDPs and multidomain proteins, highlights the value of understanding how chemical probes broadly affect a conformational landscape.

High-throughput small-angle X-ray scattering (HT-SAXS) efficiently profiles target–ligand complexes and their structural ensembles in solution^7–9^ and is adaptable to a broad range of biomolecular targets^2,10,11^. Without molecular weight and labeling constraints, SAXS monitors solution architectures, which can differ from crystallographic forms, and readily complements solution conditions used by other ligand discovery screens. Advances in sample handling and data processing at SAXS synchrotron beamlines^12,13^ have also increased access to high-quality HT-SAXS target–ligand datasets for academic users.

Importantly, SAXS is sensitive to a variety of conformational changes on multiple distance scales. Although often considered a low-resolution technique, SAXS curves report the entire distribution of biomolecular interatomic distances. Thus, small local changes in molecular architecture are detected as shifts in long-range interatomic distances, which are sensitively measured in the low- and mid-angle regions of the SAXS curve. This allows monitoring of order–disorder transitions, domain rotations, intramolecular expansion and contraction, formation of biomolecular assemblies and other conformational changes^14,15^. Thus, the translatability, applicability and conformational sensitivity of HT-SAXS make it well suited for screening target–ligand conformations during discovery and triaging fragment hits by conformational and functional impact.

Time-resolved experiments can further broaden the impact of HT-SAXS during fragment discovery by monitoring multiple target states in a single experiment^16^. TR-SAXS experiments have tracked changes to biomolecular conformation and dynamics triggered by a variety of stimuli, including temperature, pressure, pH, light and chemical ligands. TR-SAXS measurements are also uniquely positioned to probe redox-responsive targets, as intrinsic X-ray exposure drives target conversion from oxidized to X-ray-stimulated reduced states. Such redox-responsive systems include flavoenzymes^17^, regulatory redox-active disulfides^18^, metalloregulatory proteins^19^ and photosensors such as cryptochromes^20^. Thus, incorporating conformational triggers into HT-SAXS experimental design and capitalizing on synchrotron detector speeds can reveal how ligands interact with multiple unique target states and influence conformational evolution during fragment screening experiments.

Here, we unveil, test and validate a library-screening approach that integrates biophysical ligand screening and TR-HT-SAXS to identify early fragment chemotypes targeting distinct allosteric redox states of the exemplary mitochondrial protein AIF^21–23^. A partner of protein import chaperone CHCHD4/MIA40, AIF regulates the biogenesis of mitochondrial oxidative phosphorylation (OXPHOS) complexes, an area of increased focus for cancer therapeutics^24,25^. A 60-kDa FAD-dependent oxidoreductase, AIF allosterically switches between two distinct redox states. NADH binding by the oxidized AIF monomer reduces its FAD cofactor at a centralized active site, allosterically disrupting hydrogen bond networks to release a 50-residue surface loop (C-loop) and stimulate dimerization at the AIF surface^21,26^. Oxidized NAD^+^ and reduced FADH^–^ form a long-lived charge-transfer complex (CTC), which stabilizes and maintains the reduced dimeric state of AIF. NADH-activated AIF dimers support import of OXPHOS subunits by CHCHD4 and are critical for mitochondrial function^21–23^. Many disease-causing AIF mutations in humans destabilize the AIF dimer and result in mitochondrial defects^27,28^, pointing to the value of treatments that selectively target this allosteric state.

Using TR-HT-SAXS screening, we monitored fragment impacts on both oxidized and X-ray-reduced AIF states, identifying unique fragment chemotypes selectively targeting the AIF monomer or stimulating the OXPHOS-competent dimer. Kinetic analysis of AIF monomer–dimer transitions, as monitored by the volatility-of-ratio metric (*k*_VR_), additionally delineates key functional groups that support or hinder dimerization, demonstrating that TR-HT-SAXS kinetics can usefully probe structure–activity relationships (SAR) during early fragment discovery. We show that the top-ranked class of 4-aminoquinolines from the SAXS screen stabilize AIF dimerization and stimulate binding to mitochondrial partner CHCHD4. Crystallographic analysis of AIF–aminoquinoline complexes reveals that this chemotype selectively engages the reduced AIF active site in a manner similar to NAD(H) but does not directly engage active site residue H454, an allosteric touchstone for AIF dimerization. Instead, the aminoquinoline scaffold displaces neighboring residue F482, which aromatically secures and maintains H454 in a dimeric configuration. Biochemical analysis of an F482 AIF mutant confirms an essential and underappreciated role for this residue in stabilizing the AIF dimer and establishes F482–H454 pairing as a target for the 4-aminoquinoline mechanism of action. Collectively, these results demonstrate the effectiveness of combining biophysical and TR-HT-SAXS ligand screening to identify functional redox- and conformational-specific fragment chemotypes and to provide early SAR for hit optimization.

## Results

### Fragment screening identifies small-molecule binders of AIF

**Fig. 1:**
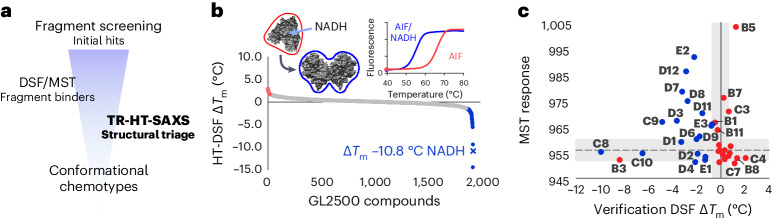
A custom fragment screen reveals unique small-molecule binders of the OXPHOS biogenesis regulator AIF. **a**, An integrated TR-HT-SAXS fragment screening workflow. **b**, Ranked HT-DSF screening of the GL2500 fragment library identifies 39 hits elevating (red) or lowering (blue) *T*_m_ with | Δ*T*_m_ | > 3 s.d. over DMSO controls. See also Supplementary Tables 1 and 2. Left inset, NADH redox binding in the AIF monomer active site results in CTC formation with reduced FAD and allosteric stimulation of AIF dimerization (AIF monomer, Protein Data Bank (PDB): 4BV6; AIF dimer, PDB: 4BUR). Right inset, control AIF melting curves with DMSO. **c**, Correlation plot of secondary DSF and MST results from GL2500 hits, color coded red (*T*_m_ elevating) or blue (*T*_m_ lowering), from the original HT-DSF screen. Gray shading indicates the DMSO 3 s.d. response ranges. Hits that exhibited significant binding in at least one verification assay (labeled, 32 fragments) were examined further following TR-HT-SAXS clustering. See also Supplementary Table 2. Extended Data Fig. 1e displays an unzoomed graph with C11/C12. Source data

To identify conformational-specific fragment scaffolds targeting AIF allosteric states, we applied a SAXS-integrated library-screening approach. This included discovering fragment binders with HT differential scanning fluorescence (HT-DSF), validating hits with standard ligand binding assays (DSF and microscale thermophoresis (MST)) and triaging conformational chemotypes unique to oxidized and reduced AIF with TR-HT-SAXS (Fig. 1a).

**Extended Data Fig. 1:**
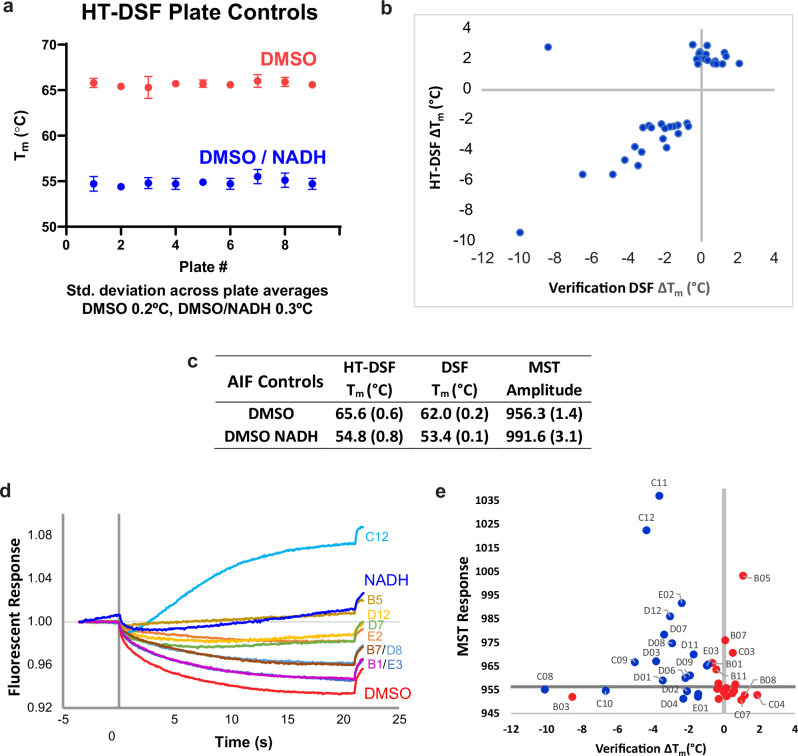
AIF HT-DSF GL2500 screening and fragment verification. **(a)** Plate-to-plate variation in AIF T_m_ with DMSO or DMSO/NADH controls. Each point represents the average of 24 wells within the indicated plate; error bars are standard deviations. **(b)** Comparison of T_m_ shifts from high-throughput and verification DSF screens for the top 39 fragments hits. **(c)** Melting temperatures and reference MST amplitudes for AIF screening controls. Standard deviations in parentheses are from 216 (HT-DSF), 6 (DSF), or 4 (MST) replicate measurements. **(d)** Exemplary MST traces from positive fragment binders. **(e)** Full-view T_m_ v MST response plot. Source data

We first screened AIF against a 2,500-fragment ‘Goldilocks’ library (GL2500)^29^ with HT-DSF (Fig. 1b, Extended Data Fig. 1a and Supplementary Table 1). The custom GL2500 library contains structurally diverse, drug-like fragments amenable to chemical elaboration. Fragments were screened at 0.75–1.5 mM against 8.3 µM AIF (~90- to 180-fold molar excess of ligand), then ranked and filtered based on significant changes in melting temperature (| Δ*T*_m_ | > 3 s.d., 1.7 °C)^30^. Native NADH ligand lowers the AIF *T*_m_ (ref. ^31^) (Fig. 1b, inset, Δ*T*_m_ = –10.8 °C), presumably from allosteric disruption of hydrogen bonds linking the AIF active site to the dimerization interface and C-loop contact site^21^. Thus, fragments with a Δ*T*_m_ <0 were also considered for their potential to induce AIF allostery directly (like NADH) or indirectly by stabilizing heat-induced disruptions to AIF hydrogen bonds. This yielded hit rates of 1.0% (*T*_m_ elevating) and 2.4% (*T*_m_ lowering) across all GL2500 fragments. The top 19 and 20 fragments from each category (39 total) were selected for further characterization (Supplementary Table 2).

GL2500 hits were evaluated by a second round of DSF and then MST to evaluate binding reproducibility (Fig. 1c). Over three-quarters of the hits showed positive binding by either DSF or MST, and one-third reproduced in both assays (14 fragments) (Fig. 1c, Extended Data Fig. 1b–e and Supplementary Table 2), resulting in 32 fragment binders of AIF.

### TR-HT-SAXS triages hit conformation and chemotype

In addition to verifying AIF binding, the DSF and MST results also supported an absence of protein aggregation among AIF–fragment complexes, critical for ensuring high-quality SAXS data. Thus, we proceeded to measure TR-HT-SAXS for AIF incubated with the 32 verified fragment hits and to assess the conformational impact on the oxidized and X-ray-reduced protein. The seven nonverified fragment hits were also included in the SAXS screening as negative controls.

**Extended Data Fig. 2:**
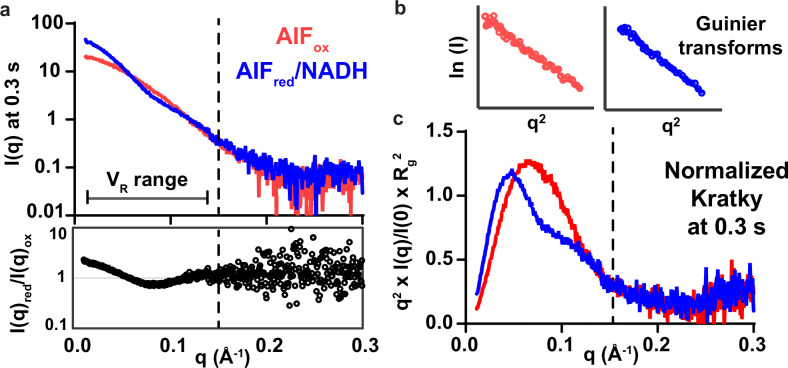
HT-SAXS DMSO benchmarks. **(a)** SAXS scattering curves (300-ms exposure) and analysis of oxidized AIF monomer (red) and NADH−reduced AIF dimer (blue). The ratio of the scattering intensities (lower panel) highlights the uniqueness of the SAXS profiles for the two allosteric states. The q-range (0.015 – 0.15 Å-1) used for *V*_R_ similarity analysis is indicated by the dotted line. **(b)** Linear Guinier transforms of the SAXS curves indicates an absence of aggregation. See also Supplementary Table 3. **(c)** Plots of normalized Kratky transforms. Source data

The AIF oxidized monomer (AIF + DMSO) and reduced dimer (AIF + NADH + DMSO) states exhibited distinct X-ray scattering profiles and parameters under the selected screening conditions, providing clear reference points for analyzing AIF–fragment samples^7^ (Extended Data Fig. 2a–c and Supplementary Table 3). To quantify statistical resolvability of AIF monomer and dimer SAXS profiles for the short 300-ms exposures applied during time-resolved data acquisition, we computed *Z-*factors for radius-of-gyration (*R*_g_) and extrapolated zero-angle scattering intensity (*I*(0)) values from independent replicate measurements^7^. This yielded 0.64 (*R*_g_) and 0.85 (*I*(0)), where *Z*-factors 0.5–1.0 provide effective discrimination between maximum signal and baseline states.

SAXS samples with compound-matched buffers and controls were assembled in 96-well plates with HT-DSF fragment concentrations (0.5–1 mM) and 4 mg ml^–1^ AIF (~8- to 16-fold molar excess of ligand), emphasizing the translatability from ligand binding assay to HT-SAXS assay. TR-SAXS data were collected in batch format following benchmarked protocols for chemical ligand screening^7^ with 300-ms framing of a 10-s exposure using the unique HT-SAXS platform developed at the SIBYLS SAXS Synchrotron Beamline (BL12.3.1, sibyls.als.lbl.gov)^9,10,12^.

**Fig. 2:**
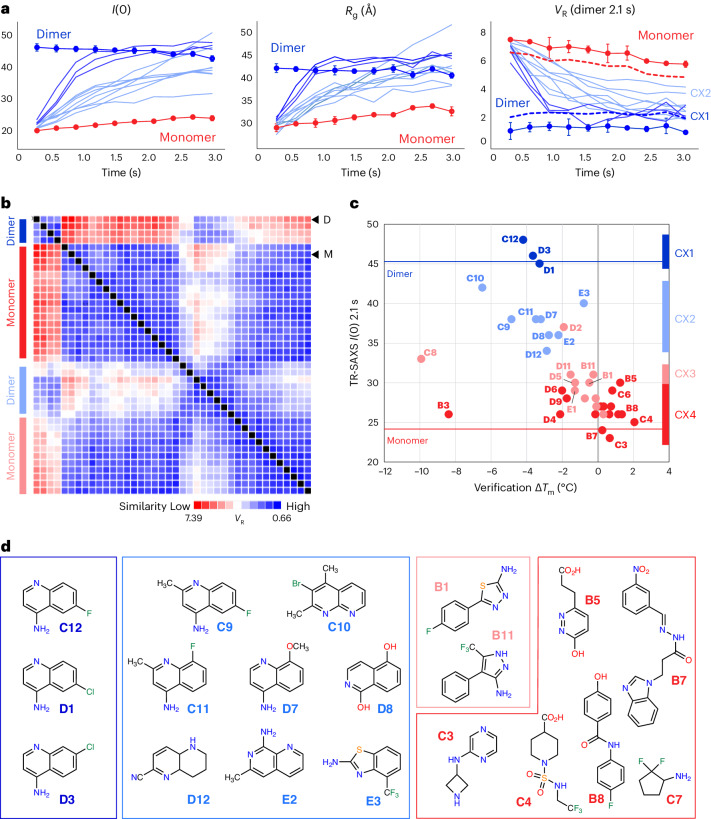
TR-HT-SAXS triages hits by conformational impact and fragment chemotype. **a**, *I*(0), *R*_g_ and *V*_R_ (AIF–NADH–DMSO dimer reference) values from select time-resolved GL2500 SAXS datasets compared to AIF monomer (connected red circles) and NADH dimer (connected blue circles) DMSO benchmarks. GL2500 curves are colored by similarity cluster from **b**: dark blue, CX1; light blue, CX2. Error bars for AIF monomer and dimer controls represent standard deviations from two independent replicates (three replicates for AIF–NADH *I*(0) and *R*_g_). Dotted lines represent AIF monomer and dimer *V*_R_ significance thresholds of three times the average standard deviation across the time series. **b**, Clustered GL2500 SAXS *V*_R_ similarity matrix at 2.1 s. High similarity is indicated in blue, whereas low similarity is indicated in red according to the *V*_R_ scale bar. Arrows indicate the row position of AIF dimer (D) or monomer (M) controls. **c**, Extrapolated *I*(0) values from 2.1-s GL2500 SAXS datasets plotted against Δ*T*_m_ from the secondary DSF assay. Data points are color coded by SAXS similarity cluster from **b**. **d**, GL2500 similarity clusters reveal SAXS-triaged chemotypes. Displayed CX3 and CX4 fragments exhibited the greatest response in the DSF and MST verification assays: dark blue, CX1; light blue, CX2; light red, CX3; dark red, CX4. Source data

**Extended Data Fig. 3:**
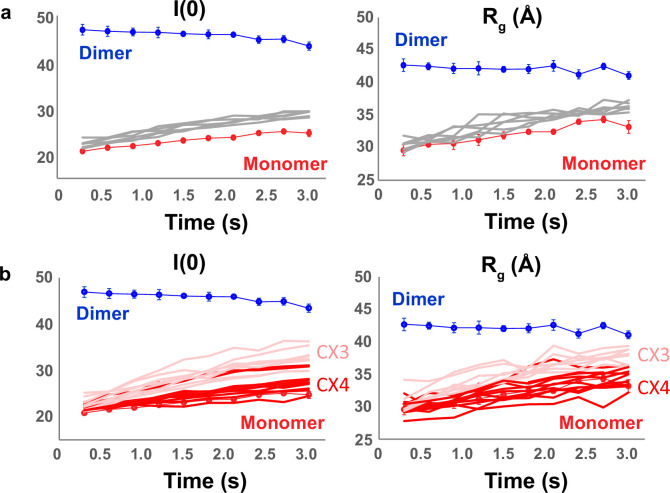
Time-evolved SAXS parameters for non-reproducing and non-dimerizing hits. **(a)** Plots of time-evolved I(0) and R_g_ values for non-reproducing hits **B2**, **B4**, **B6**, **B9**, **B10**, and **B12** compared to AIF-DMSO (red circles) and AIF-NADH_−_DMSO controls (blue circles). Errors bars for AIF-DMSO represent the standard deviation of 2 independent replicates; error bars for AIF-NADH^−^DMSO represent the standard deviation of 3 independent replicates. **(b)** Plots of time-evolved I(0) and R_g_ values for SAXS chemotype clusters CX3 (pink) and CX4 (red) compared to AIF-DMSO (red circles) and AIF-NADH-DMSO (blue circles) controls. Errors bars for AIF-DMSO represent the standard deviation of 2 independent replicates; error bars for AIF-NADH-DMSO represent the standard deviation of 3 independent replicates. Source data

Buffer-corrected scattering profiles from the earliest SAXS time point and corresponding Guinier transforms were visually assessed to eliminate samples exhibiting poor background subtraction or aggregation. A single dataset (**C5**) was excluded due to bubble formation in the sample cell, leaving 38 AIF–ligand datasets (32 verified and 6 nonverified binders). We analyzed time-resolved *R*_g_ and molecular-weight-related *I*(0) values to generate a first-order assessment of ligand engagement of oxidized and X-ray-reduced AIF. Time-evolved *I*(0) and *R*_g_ values from the six control fragments track closely with the AIF–DMSO control (Extended Data Fig. 3a). Time series data from the 32 verified fragments separated into two subsets: fragments that exhibit early time-dependent increases in *I*(0) and *R*_g_, consistent with X-ray-stimulated AIF dimerization (Fig. 2a), and fragments that exhibit no or moderate time evolution without signs of dimerization (Extended Data Fig. 3b).

The AIF–DMSO monomer control exhibited a modest rise in *R*_g_ (3–4 Å) at later time points, whereas the AIF–NADH–DMSO benchmark maintained consistent *I*(0) and *R*_g_ values over the full time series. The small change in *R*_g_ observed for the AIF–DMSO time series is reminiscent of previous SAXS observations for obligately monomeric AIF mutants treated with NADH^21^. Defective for dimerization, these AIF mutants still release the C-loop insert from the protein surface in response to NADH reduction, resulting in a limited *R*_g_ increase. The comparable change in the AIF–DMSO control suggests that prolonged X-ray exposure may stimulate a similar response. Collectively, the AIF monomer and dimer benchmarks indicate that intrinsic X-ray exposure neither robustly stimulates nor disrupts dimerization. Thus, the observed time-dependent transitions mimicking the AIF dimer are selectively stimulated by the presence of specific GL2500 fragments.

To establish that these ligand-induced changes are not artifactual and reflect canonical conformational transitions, we calculated SAXS *V*_R_^32,33^ values between time-evolved AIF–fragment scattering curves and time-matched AIF dimer DMSO benchmarks (Fig. 2a, right). *I*(0) and *R*_g_ are derived from signal of the low-*q* region, reflecting the largest molecular distances within the total interatomic distribution. By contrast, the *V*_R_ metric incorporates scattering intensities across an extended *q-*range (here, 0.015–0.15 Å^–1^) and leverages the full conformational fingerprint across multiple distance scales to evaluate similarity between paired SAXS curves. Thus, *V*_R_ analysis is well positioned to distinguish if fragments enable biologically relevant AIF dimerization or stabilize the AIF monomer.

The corresponding time-resolved *V*_R_ plots from responsive AIF–fragment complexes revealed that their scattering similarity increases (*V*_R_ decreases) relative to the AIF dimer benchmark, mirroring the time dependence observed for *I*(0) and *R*_g_ (Fig. 2a). Likewise, *V*_R_ values for independent AIF monomer and dimer benchmarks reflect the stability of their time-resolved *I*(0) and *R*_g_ values. Mirroring the trend in *R*_g_, later AIF monomer time points also showed a modest decrease in *V*_R_. To verify the ability of *V*_R_ to differentiate changes in scattering signals from short millisecond exposures, standard deviations from replicate AIF monomer and dimer benchmarks were used to monitor intrinsic variation in the *V*_R_ metric and to define a significance threshold (three times the average standard deviation of each benchmark time series) (Fig. 2a). *V*_R_ values from responsive AIF–fragment complexes progressively fell below the *V*_R_ monomer threshold, and a more focused subset entered the significance threshold of the AIF dimer. Thus, select GL2500 fragment binders appear to enable canonical AIF dimerization.

**Extended Data Fig. 4:**
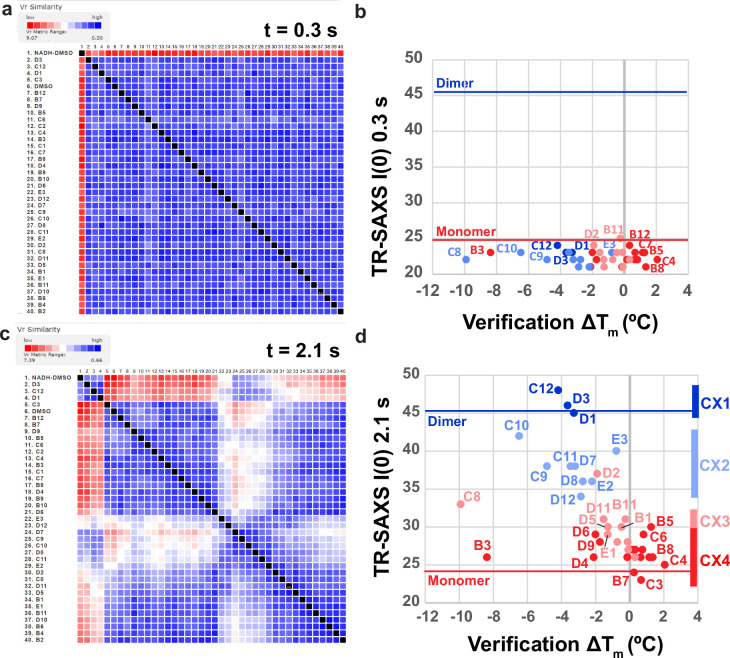
SAXS similarity clustering for oxidized and reduced AIF. **(a)** Clustered SAXS similarity matrix of oxidized AIF-fragment samples at 0.3 s. High similarity is indicated in blue, while low similarity is indicated in red according to the *V*_R_ scale bar. High similarity to the AIF-DMSO control across the matrix indicates GL2500 fragments do not perturb oxidized, monomeric AIF architecture. **(b)** Extrapolated zero-angle scattering intensities I(0) from the 0.3-s time point are plotted against shifts in melting temperature measured by DSF. The I(0) values are consistent with an absence of aggregation. Data points are color-coded by SAXS similarity cluster from Fig. 2b. **(c)** Clustered SAXS similarity matrix of reduced AIF-fragment samples at 2.1 s. High similarity is indicated in blue, while low similarity is indicated in red according to the *V*_R_ scale bar. Reproduced from Fig. 2b to display fragment identifiers next to the matrix. **(d)** Extrapolated zero-angle scattering intensities I(0) from the 2.1-s time point are plotted against shifts in melting temperature measured by DSF. Data points are color-coded by SAXS similarity cluster from Fig. 2b. Reproduced from Fig. 2c. Source data

Assembly of *V*_R_ values into SAXS similarity matrix (SSM) maps^32,33^ allows clustering and ligand ranking to identify chemical matter associated with conformational chemotypes. Using the SAXS Similarity web application (https://sibyls.als.lbl.gov/saxs-similarity/), we first applied *V*_R_ clustering analysis to all 38 AIF–fragment samples for the earliest SAXS time point (0.3 s) to assess fragment impact on the structure and stability of oxidized AIF before X-ray-induced reduction (Extended Data Fig. 4a,b). The AIF–fragment complexes share high similarity with the AIF monomer scattering benchmark, demonstrating that the fragment hits are not structural destabilizers and do not cause dimerization of oxidized AIF. The fragments may induce subtler structural changes captured by intensity information at higher resolutions (*q* > 0.15 A^–1^). However, the short millisecond framing of the time-resolved exposures limits signal-to-noise in this region. Increasing sample concentrations or averaging data between replicate samples could offer further options for accessing SAXS signal at these higher *q-*values.

We next applied *V*_R_ clustering to the extended time series (0.6–3.0 s) and discovered a progressive partitioning of fragment effects on X-ray-reduced AIF (Supplementary Fig. 1). Because transitions in many time-resolved *I*(0) values reach a maximum at 1.8–2.1 s (assessed by exponential fitting), we chose to focus initial *V*_R_ calculations and clustering analysis on AIF–NADH–DMSO scattering at 2.1 s. The *V*_R_ clustering captured four distinct ligand groups, reflecting strong (SAXS cluster 1 (CX1)), moderate (CX2), weak (CX3) or absent (CX4) similarity relative to dimerized AIF (Fig. 2b and Extended Data Fig. 4c,d). Superimposing similarity classifications with corresponding molecular-weight-related *I*(0) values and *T*_m_ shifts from the secondary DSF screen showed that CX1 and CX2 fragments stimulate a rise in molecular weight and reduction in *T*_m_ similar to the AIF native NADH ligand (Fig. 2c). We verified that these changes did not arise from aggregation by verifying linearity of Guinier transforms from scattering curves at 0.3-s and 2.1-s time points (Supplementary Figs. 2–6).

Inspection of clustered chemical scaffolds from verified binders demonstrates that SAXS similarity analysis successfully triages fragment hits into unique conformational chemotypes. The strongly dimerizing CX1 cluster is exclusively populated by halogenated 4-aminoquinolines (Fig. 2d, dark blue). The broader, moderately dimerizing CX2 cluster is likewise enriched with aminoquinolines and 6,6-fused heterocycles, which exhibit a wider diversity of functionalization (Fig. 2d, light blue). By contrast, the monomeric CX3 and CX4 clusters are enriched in linked 5- and 6-membered heterocycles (Fig. 2d, light and dark red, respectively). Thus, the SAXS analysis partitions chemical scaffolds by architectural impact on AIF and allows for further hit characterization according to conformational class.

**Extended Data Fig. 5:**
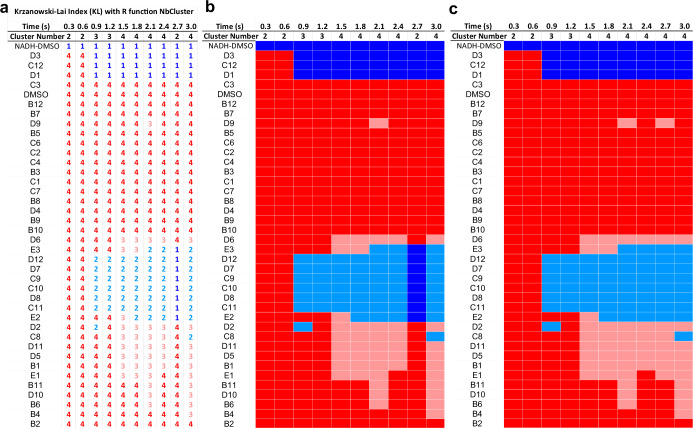
K-means clustering of time-resolved SAXS V_R_ SSMs. **(a)** Time-resolved *k*-means cluster numbers and assignments for AIF-fragment complexes across the time series (CX1, blue; CX2, light blue; CX3, pink; CX4, red) (*left*). Cluster number at each time point was determined by maximizing the Krzanowski-Lai Index (KL)^55^ using the *NbClust* function in R^53,54^. **(b)** Heat-map representation of clusters in Panel a. **(c)** Heat map is identical to that in Panel b, but with a 4-cluster assignment shown for t = 2.7 s calculated with the *kmeans* function in R. Source data

To cross-verify the clustering efficiency of the web application, which sorts paired *V*_R_ Euclidean distances with a single-linkage agglomerative hierarchical clustering (AHC) algorithm, we also clustered each *V*_R_ SSM dataset independently using a *k*-means approach and examined evolution of the clustering across the time series (Extended Data Fig. 5). The *k*-means approach captures the four AHC similarity clusters, revealing early emergence and progressive growth of CX1 and CX2 with later appearance of CX3. Elevated variation in the sorting between *k*-means CX3 and CX4 at later time points suggests a lower barrier between these clusters and is consistent with the similar linked heterocycle scaffolds populating both groups.

### TR-SAXS kinetic analysis extends SAXS chemotype SAR

**Fig. 3:**
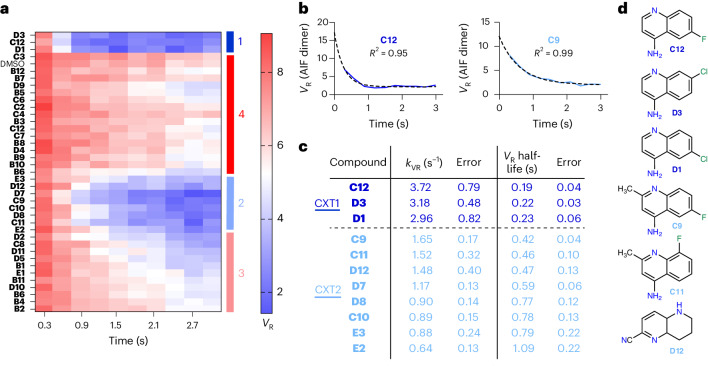
TR-SAXS kinetic analysis extends SAXS chemotype SAR. **a**, GL2500 SAXS *V*_R_ heat map capturing time-resolved *V*_R_ values of AIF–ligand complexes calculated with reference to the AIF dimer benchmark at 2.1 s. High similarity is indicated in blue, whereas low similarity is indicated in red according to the *V*_R_ scale bar. Clustered chemotype classes (CX1–CX4) described in Fig. 2 are indicated on the right. **b**, Exemplary exponential fits to *V*_R_ transition curves for AIF–**C12** and AIF–**C9** complexes. **c**, CXT1 (dark blue) and CXT2 (light blue) compounds kinetically ranked and clustered by *V*_R_ transition rates, *k*_VR_. **d**, The 4-aminoquinoline scaffold stimulates the fastest dimer transition rates among the GL2500 hits. Source data

Clustering analysis of similarity matrices from individual SAXS time points successfully identifies dimeric AIF chemotypes. However, the unique time evolution of each SAXS chemotype cluster suggests that fragment SAR may also affect the rate of AIF conformational transitions. Thus, we explored whether further structure–activity information could be derived from kinetic analysis of *V*_R_ transition rates. A heat map of the entire time-resolved *V*_R_ dataset (with each *V*_R_ value calculated relative to the AIF–NADH–DMSO scattering curve at 2.1 s) revealed that the strongly dimerizing CX1 cluster exhibits the fastest *V*_R_ transition rates, followed by those of the moderately dimerizing CX2 group (Fig. 3a). We proceeded to quantify these kinetic transitions by calculating first-order rate constants for the CX1 and CX2 *V*_R_ transition curves (*k*_VR_). Overlaying exponential fits with the SAXS *V*_R_ values indicates that the conformational transition of AIF is well described by this relationship (Fig. 3b and Supplementary Fig. 7). We ranked the *k*_VR_ transition rates, clustered by AHC and *k*-means approaches and examined the corresponding scaffolds (Fig. 3c,d). AHC and *k*-means clustering returned identical clusters (CXT1 and CXT2) that overlay with those derived from the SSM clustering analysis. The *k*_VR_ ranking highlighted enrichment of 4-aminoquinolines, which compose six of the top seven fragments, supporting the 4-aminoquinoline scaffold as the fundamental allosteric ligand. The kinetic ranking further differentiates specific SAR across 4-aminoquinoline fragments. Halogen substitutions at positions 7 and 8 (fragments **C12**, **D1** and **D3**) optimally accelerate dimerization. By contrast, methyl substitution at position 3 (fragments **C9** and **C11**) and methoxy substitution at position 6 (fragment **D7**) slow allosteric transition. Thus, the kinetic analysis of *V*_R_ transition rates corroborates the single-point SAXS conformational clustering and provides relevant SAR for allosteric ligands.

With the consistent selection of 4-aminoquinolines among dimer-stimulating fragments, we also examined 4-aminoquinolines excluded from the original GL2500 hits to provide counter SAR. Four 4-aminoquinolines were identified (Supplementary Fig. 8). Three of these incorporated charged (carboxylate) or large (bromine) functional groups adjacent to the primary amine. Their exclusion suggests that unfavorable position 2 substitutions may interfere with important protein contacts formed by the primary amine. The fourth fragment is an unmethylated analog of **C11**, placing a fluorine at position 6 (**G4**; Supplementary Fig. 8). The similarity of this fragment to the top kinetically ranked fragments indicates that additional structural insight is needed to understand its exclusion.

### Top-ranked SAXS chemotype stimulates AIF binding to CHCHD4

**Fig. 4:**
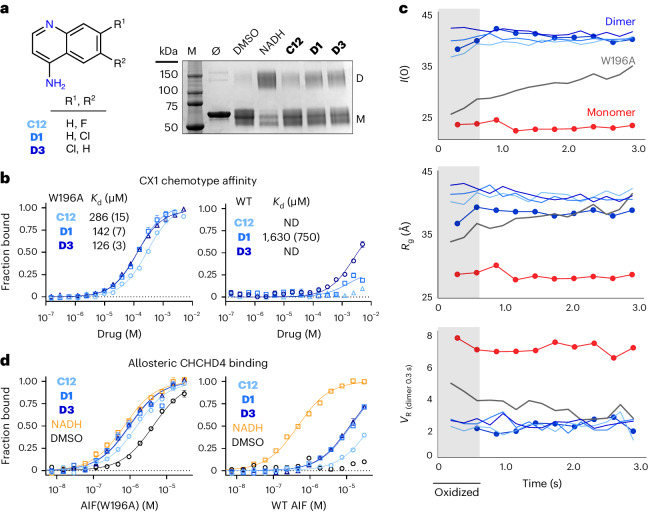
The top-ranked SAXS chemotype selectively binds dimeric AIF and stimulates CHCHD4 binding. **a**, Left, scheme for the CX1 4-aminoquinoline scaffold. Right, CX1 fragments induce weak AIF dimerization captured by bis(sulfosuccinimidyl)suberate (BS^3^) amine cross-linking; M, molecular weight marker lane. The displayed gel is representative of three independent experiments. **b**, CX1 fragments selectively bind dimer-permissive Atto488-labeled AIF(W196A) (left) over the wild-type (WT) AIF monomer (right) by MST. Standard errors of fitting are included in parentheses. Each curve represents the average of three thermophoresis scans of a representative binding titration; error bars represent standard deviations ND, affinity not determined. **c**, TR-SAXS analysis of AIF(W196A)–CX1 complexes. SAXS parameters for AIF(W196A) saturated with **C12** (light blue), **D1** (medium blue), **D3** (dark blue) or DMSO (gray) are plotted with wild-type AIF monomer (red circles) and dimer (blue circles) DMSO controls. *V*_R_ is calculated relative to the initial exposure of dimeric AIF–NADH. The light gray region highlights exposures containing oxidized W196A protein (*t* = 0.2 s, 0.4 s) before X-ray-induced FAD reduction. **d**, CX1 fragments stimulate binding between Atto488-labeled mitochondrial partner CHCHD4 and wild-type AIF (right) or AIF(W196A) (left). See Supplementary Table 4 for binding affinities. MST binding buffer in these experiments was supplemented with 5 mM TCEP to prevent CHCHD4 intermolecular disulfides. Each curve represents the average of three thermophoresis scans of a representative binding titration; error bars represent standard deviations. Source data

To verify conformational chemotyping provided by the SAXS fragment screen, we selected the CX1 SAXS chemotype for further biochemical and structural analysis. Cross-linking tests for AIF dimerization without X-rays revealed that CX1 fragments stimulate weak dimerization of oxidized AIF (Fig. 4a). This points to a structural, rather than a direct redox-mediated, mechanism of action. Past studies have shown that exogenous reduction of the AIF FAD cofactor configures the protein for dimerization, but concurrent binding by NAD^+^ ligand is required to allosterically stabilize the dimeric state^34,35^. X-ray-induced reduction is expected to ‘unlock’ AIF in a similar manner, suggesting that CX1 fragments exert their effect by structurally engaging this activated, dimer-permissive state. To test this, we investigated whether CX1 fragments could interact with the preconfigured dimer-permissive AIF(W196A) mutant, an allosteric intermediate that dimerizes without NADH in a concentration-dependent manner^21^. All three CX1 fragments preferentially bound oxidized AIF(W196A) with the micromolar affinities expected of screening hits (Fig. 4b). The CX1 fragments exhibited weaker interaction with wild-type AIF, consistent with the cross-linking experiments. That CX1 fragments can stabilize a small dimeric population of wild-type AIF suggests that the oxidized protein may transiently sample an activated state without external stimulus.

We next measured the ability of CX1 fragments to enable and sustain AIF(W196A) dimerization in TR-SAXS experiments (Fig. 4c and Supplementary Fig. 9). At protein concentrations used for SAXS, oxidized AIF(W196A) exists as a mixture of monomer and dimer species in the absence of ligand. Progressive photoreduction by X-rays shifts this equilibrium toward the dimeric state, as reflected by a rise in *I*(0) and *R*_g_ and a decline in *V*_R_ relative to AIF dimer controls (Fig. 4c). When the AIF(W196A) mutant is co-incubated with CX1 fragments, the oxidized mutant fully dimerizes and remains dimeric throughout the time series, similar to the wild-type AIF–NADH–DMSO control. The ability of the CX1 chemotype to successfully engage and shift an allosteric intermediate of AIF’s switching pathway supports these fragments as true conformational effectors.

To verify that the CX1 chemotype enables a biologically functional AIF dimer, we measured binding between ligand-bound AIF and mitochondrial binding partner CHCHD4, a critical protein import chaperone for respiratory complex components. Mitochondrial persistence of CHCHD4 is linked to interaction with NADH-activated AIF^22^, and respiratory complex defects have been observed in individuals with AIF disease-causing mutations^28^. Binding measurements by MST establish that interaction between wild-type AIF and Atto488-labeled CHCHD4 occurs only in the presence of NADH, consistent with previous reports^22^ (Fig. 4d). CX1 fragments stimulates binding between wild-type AIF and CHCHD4 but at reduced affinity. This aligns with the weak fragment engagement by the wild-type protein. Measuring CHCHD4 binding to dimer-permissive AIF(W196A) reveals that this mutant is additionally capable of engaging CHCHD4 in the absence of NADH ligand, although at lower affinity than the wild-type protein, aligning with other reports^36^ (Fig. 4d, left, and Supplementary Table 4). This is consistent with a mixture of active AIF(W196A) dimers and inactive AIF(W196A) monomers in the binding reaction. Addition of NADH, expected to shift AIF(W196A) completely to the dimer state, stimulates wild-type binding affinity between CHCHD4 and the W196A mutant. Notably, the CX1 fragments also stimulate CHCHD4 binding to the AIF(W196A) mutant at affinities comparable to NADH (Supplementary Table 4), demonstrating their ability to enable a biologically functional AIF dimer and modulate a key physiologic target. Together, these results confirm the CX1 chemotype as a conformational effector and suggest that CX1 fragments leverage an allosteric mechanism of action similar to NADH.

### Aminoquinoline and NAD^+^ allosterically stabilize AIF dimers

**Fig. 5:**
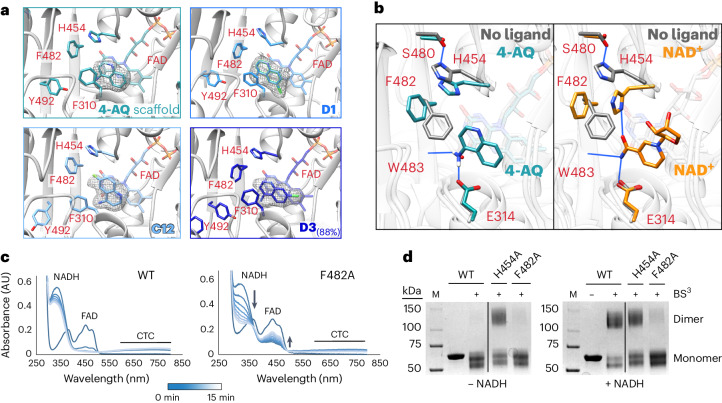
Crystallographic structures reveal how aminoquinolines and native NAD^+^ ligands allosterically stabilize the AIF dimer. **a**, CX1 fragments (**C12**, **D1** and **D3**) and 4-aminoquinoline (**4AQ**) target the AIF active site when soaked into AIF(W196A) crystals (2.38–2.6 Å). Polder maps are displayed at 5*σ*, 1.5–2.0 Å around the ligand surface. See also Extended Data Fig. 6 and Supplementary Tables 5–7. **b**, Superposition of AIF(W196A)–**4AQ** (teal) and native dimeric AIF–NAD(H) (yellow, PDB: 4BUR) complexes with ligand-free AIF monomer (gray, PDB: 4BV6). Hydrogen bonds are indicated by blue lines. The NAD^+^ ligand has been trimmed for clarity. **c**, Time-evolved UV-Vis absorbance spectra of wild-type AIF and AIF(F482A) monitoring FAD reduction and CTC formation stimulated by NADH. Spectra were collected every 2.5 min for 15 min after addition of NADH. AU, absorbance units. **d**, SDS–PAGE of amine cross-linking reactions of wild-type AIF, AIF(H454A) and AIF(F482A) in the absence and presence of NADH. The displayed gels are representative of three independent experiments. Two intervening lanes between wild-type AIF and AIF(H454A) (lanes 3 and 4) have been removed for clarity. Full images of the SDS–PAGE gels are displayed in Supplementary Fig. 11. Source data

**Extended Data Fig. 6:**
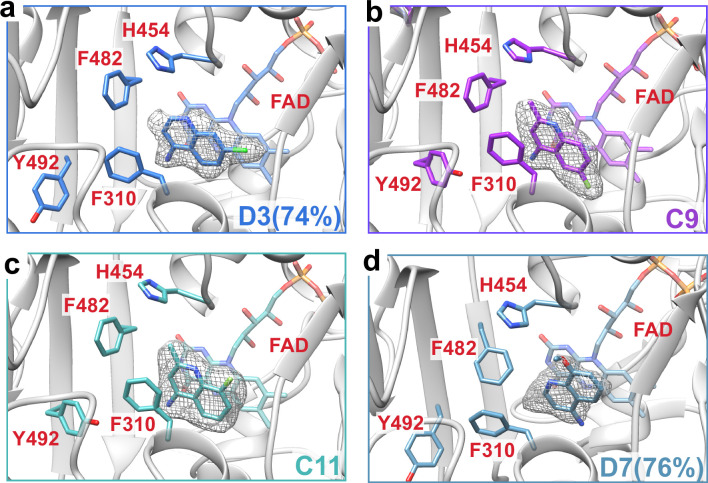
Crystallographic Structures of Wild-type AIF-aminoquinoline Complexes. Zoomed views of AIF’s active site with Polder maps displayed at 5σ within 1.5-2.0 Å of CX1 ligand **(a) D3** (2.25 Å, Monomer A, 74% ligand occupancy) and CX2 ligands **(b) C9** (2.4 Å, Monomer A), **(c) C11** (2.3 Å, Monomer B), and **(d) D7** (2.25 Å, Monomer A, 76% ligand occupancy). See Supplementary Fig. 10 for simulated-annealing composite omit maps of ligands and active site residues. Crystallographic collection and refinement statistics are reported in Supplementary Tables 6-7.

To understand how SAXS CX1 and CX2 chemotypes engage AIF and enable dimerization, we crystallized select fragment hits with the W196A dimer-permissive mutant and wild-type AIF (Fig. 5a,b, Extended Data Fig. 6a–d, Supplementary Fig. 10 and Supplementary Tables 5–7). Crystal structures of the W196A mutant bound to CX1 fragments and the underlying 4-aminoquinoline (**4AQ**) scaffold revealed that these scaffolds localize to the NADH active site, mirroring the native NADH ligand (Fig. 5a), as do CX1 and CX2 scaffolds bound to the wild-type protein (Extended Data Fig. 6a–d). In all four W196A structures, the CX1 quinoline core aromatically pairs with the AIF FAD cofactor, positioning the primary amine to hydrogen bond with the E314 side-chain carboxylate and the W483 backbone carbonyl. Similar aromatic and hydrogen bond contacts are made by the NAD(H) nicotinamide amine^26,37^ (Fig. 5b). To accommodate this amine interaction, the aromatic side chain of F482 must rotate out of the binding site. Aligned with this, AIF structures with CX1 and CX2 ligands exhibiting partial occupancy indicate mixed orientations of the F482 aromatic ring (Supplementary Fig. 10).

Unlike the native nicotinamide, the CX1 aminoquinolines do not hydrogen bond with active site H454, an allosteric touchstone for AIF dimerization (Fig. 5b). In the ligand-free AIF monomer, H454 forms a sequestering hydrogen bond interaction with S480. AIF interaction with NAD(H) disrupts this interaction and releases H454 into the active site to engage the nicotinamide, allosterically enabling the AIF dimerization interface^21,26^. The dimer-permissive W196A mutant also allosterically releases H454 into the active site, absent NADH ligand binding^21^. This points to H454 release as a preconfiguring event that allows CX1 fragments to engage the AIF active site and stabilize the dimer. In AIF structures with NADH and CX1 fragments, the released H454 imidazole ring is well positioned to aromatically contact and retain the displaced phenylalanine ring of F482, thus allowing access to W483 and E314 contact sites (Fig. 5b). That the CX1 fragments interact weakly with oxidized AIF monomer suggests that H454 availability is necessary for full F482 displacement and subsequent aminoquinoline binding. These structural results would thus explain CX1 and CX2 chemotype selectivity for reduced dimeric AIF.

The ability of the CX1 chemotype to shift the dimer-permissive W196A mutant toward the dimeric state also implicates F482 displacement and H454–F482 pairing in allosterically stabilizing the AIF dimer. To test the role of this interaction in maintaining AIF dimers, we assayed the AIF point mutant F482A for its ability to stably engage NADH and form dimers. Following NADH reduction, the reduced FADH^–^ and oxidized NAD^+^ of AIF form a stable CTC detectable by spectroscopy. We monitored the ability of wild-type AIF and the F482A mutant to form stable CTCs with NADH using UV-Vis absorbance spectroscopy (Fig. 5c). In the presence of excess NADH, the absorbance signature from the AIF FAD cofactor is quenched, while a corresponding absorbance rise at 600–800 nm signifies robust CTC formation and dimerization. By contrast, although the F482A mutant shows early signs of FAD reduction and CTC formation, it exhibites progressive reoxidation of the FAD cofactor, a decrease in CTC absorbance signal and continued depletion of excess NADH. This indicates a destabilized F482A CTC complex with poor retention of allosterically enabling NAD^+^ ligand. Previous studies captured a similar effect with mutation of allosterically enabling H454, which fails to form the CTC and instead rapidly oxidizes NADH pools^21,26^. Cross-linking assays also indicate weak maintenance of the dimer by the F482A mutant compared to robust dimerization by wild-type AIF (Fig. 5d). The previously characterized H454A mutant forms an obligate dimer without NADH^21^, supporting its role as gatekeeper to AIF dimerization. Together, these results confirm an essential role for F482 in stabilizing the AIF dimer and reinforce it as a target for the CX1 and CX2 chemotypes’ mechanism of action.

**Fig. 6:**
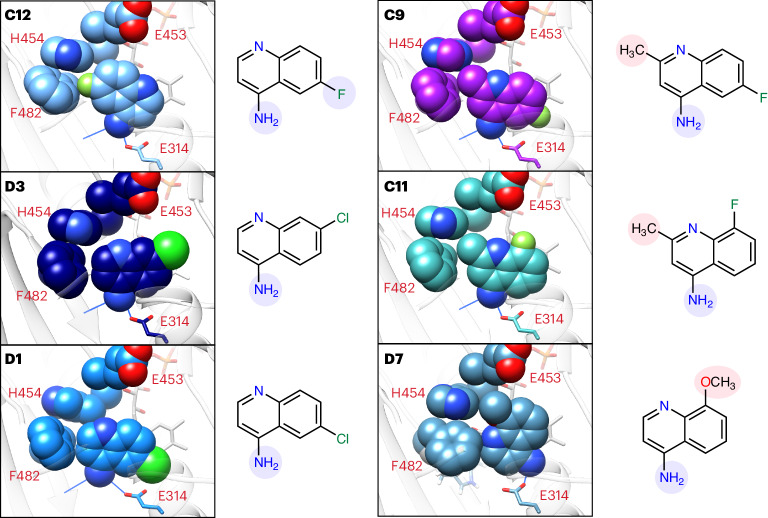
Aminoquinoline active site orientations support TR-SAXS SAR. Crystallographic complexes of CX1 and CX2 fragments with AIF(W196A) (**C12**, **D3** and **D1**) or wild-type AIF (**C9**, **C11** and **D7**) reveal stabilizing active site contacts and sources of steric interference that correlate with AIF dimerization rates. Spheres represent atomic van der Waals radii. Noncarbon atoms are colored dark blue (nitrogen), red (oxygen), light green (fluorine) and neon green (chlorine). Hydrogen bonds between the active site and primary amine are shown with blue lines. Substituents enhancing or hindering contact with the AIF active site are highlighted in blue and red, respectively, on the associated two-dimensional chemical diagrams. The images are ordered based on *k*_VR_ ranking (see Fig. 3).

The AIF–aminoquinoline structures also underscore and illuminate fragment SAR originally revealed by SAXS *V*_R_ kinetic ranking. In addition to hydrogen bonding between the primary amine and AIF active site, the structure of top-ranked fragment **C12** additionally reveals a unique halogen-π contact^38^, which places electronegative fluorine compactly against the F482 and H454 aromatic rings, reinforcing their pairing (Fig. 6). Although fluorine attraction reverses and rotates the aminoquinoline scaffold, the primary amine still hydrogen bonds with the active site. By contrast, the complementary chlorine-substituted analogs **D3** and **D1** with slower *k*_VR_ rates do not make analogous halogen-π contacts, presumably from steric restriction of the larger chlorine atom. They still form optimal amine hydrogen bonds with the active site, and the aromatic edge at positions 2 and 3 is aligned to form edge contacts with F482 and H454 aromatic rings. Introducing methyl at position 3 for fluorinated analogs **C9** and **C11** prevents formation of a **C12**-like fluorine-π contact, as the methyl (**C9**) or amine (**C11**) of the rotated scaffold will clash with L311 on the opposite side of the active site. In their observed orientation, the methyl substitutions interfere with aromatic edge contacts to F482 and H454, which presumably slows stimulated AIF dimerization. Finally, the lowest ranked **D7** aminoquinoline is rotated to minimize steric clash between its position 6 methoxy and active-site residues H454 and F482, eliminating the hydrogen bond with the W483 backbone and failing to fully displace F482 from its monomeric position.

Similarly, the structures also illuminate the counter SAR of excluded 4-aminoquinolines. The carboxylate and bromine substitutions at position 2 present a clear steric obstacle to aminoquinoline pairing with the FAD cofactor and hydrogen bonding between the primary amine and active site. The structures also suggest an explanation for why the unmethylated **C11** analog was not captured in the original screen (**G4**; Supplementary Fig. 8). Rotating this fragment to form a **C12**-like fluorine-π contact with F482 and H454 sets its primary amine to clash with L311. Aligning the fragment with **D3** or **D1** orients the fluorine to sterically encounter hydrogens of the C1′ carbon adjacent to the FAD isoalloxazine ring. These added steric obstacles may have prevented fragment capture in the original HT-DSF screen. Notably, **C11** methylation rotates the fluorinated scaffold to avoid C1′ steric interference, which may explain its successful active site engagement. These observations demonstrate that the crystallographic SAR supports and explains the ranking provided by the SAXS *k*_VR_ analysis, while providing a rationale for excluded aminoquinolines. Thus, the integrated SAXS and crystallography results provide robust validation of the aminoquinoline scaffold and SAR for further hit optimization.

## Discussion

Essential cellular functions rely on biomolecular conformations and assemblies with their related dynamic transitions. Introducing solution-based conformational screening early in fragment-based drug discovery provides an opportunity to identify and leverage chemotypes targeting unique conformational and functional states. Here, we have integrated TR-HT-SAXS ligand screening with conventional biophysical binding assays to identify chemical fragments targeting distinct allosteric redox states of mitochondrial import partner AIF. Extending the SAXS ligand screen with time-resolved observation additionally enables monitoring of oxidized and X-ray-reduced AIF states with a single screening experiment.

SAXS screening data and accompanying *V*_R_ similarity analysis established that fragment binders of AIF do not affect assembly of the oxidized protein but instead partition into three chemotypes (CX1, CX2 and CX3/CX4), which variably stimulate AIF dimerization during X-ray-induced reduction. Chemical similarities within clusters (halogenated 4-aminoquinolines, fused 6,6-heterocycles and linked 5- or 6-membered rings) underscore the ability of SAXS to triage chemical matter according to conformational impact. In addition to this single-point SAXS SSM analysis of oxidized and reduced AIF, we kinetically analyzed and triaged rates of stimulated dimerization across the time series by quantifying *V*_R_ transition rates (*k*_VR_). Ranking CX1 and CX2 *k*_VR_ values elevated 4-aminoquinolines as fragments inducing the fastest AIF dimerization. Moreover, the *k*_VR_ analysis additionally ranked substituents around the 4-aminoquinoline scaffold, indicating functional groups more favorable for AIF dimerization. This SAR was later validated and explained by crystallographic structures of AIF–aminoquinoline complexes. Thus, *k*_VR_ and kinetic analysis of SAXS datasets can serve as an important built-in source of SAR during early screening.

Combined biochemical, SAXS and crystallographic analysis of the CX1 chemotype revealed 4-aminoquinoline fragments to be true allosteric effectors of AIF. Our analyses demonstrated that CX1 aminoquinolines selectively engage a dimer-permissive form of AIF preconfigured by reduction of the FAD cofactor (X-ray exposure) or mutation of allosterically linked residues (AIF(W196A)). Like native NAD(H) ligand, the aminoquinoline scaffold stimulates and maintains AIF dimerization by binding in the active site and aromatically pairing with the FAD cofactor. Key aminoquinoline binding contacts (W483 and E314) require displacement of active site residue F482, which is facilitated by its aromatic pairing with allosteric touchstone H454. These observations show that this TR-HT-SAXS pipeline can identify ligands that remodel binding sites, similar to crystallographic approaches that have successfully yielded site-specific inhibitors^39,40^.

The AIF–aminoquinoline biochemistry and structures also unexpectedly implicate F482–H454 pairing in stabilizing AIF dimerization. We found that mutating F482 hinders the ability of AIF to dimerize and maintain a stable CTC with NAD^+^, reinforcing the underappreciated involvement of this residue in supporting allosteric dimerization of AIF. The extended lifetime and stability of the AIF CTC and dimer have been noted as signature properties compromised in many human disease-causing mutations^27,41^. Our results point to F482 as a key regulator of CTC lifetime and suggest its use as a model for investigating mechanisms and impacts of disease-related AIF mutants.

Weakened engagement of CHCHD4 and decreased mitochondrial complex I characterize many disease-causing AIF mutations^22,28^, but the molecular mechanisms underlying AIF–CHCHD4 interaction remain unknown. Importantly, aminoquinoline binding and remodeling of the AIF active site allosterically stimulates interaction with the AIF mitochondrial partner CHCHD4, demonstrating that these fragments induce physiologically relevant assemblies. The functional impact of the aminoquinoline scaffold supports its continued optimization into chemical probes exploring AIF partner interactions and mitochondrial biology. Overall, our SAXS screening identified an allosterically and functionally active chemotype against AIF suitable for further hit optimization and tool compound development.

By combining TR-HT-SAXS with biophysical ligand assays, our conformational screening pipeline establishes the accessibility and advantage of early structural insight during drug discovery. Importantly, it leverages conformational transition rates as a new class of screening data for fragment discovery and SAR profiling, enabling targeting of multistate biomolecular systems and processes. Although we focus on *V*_R_ to monitor conformational transition, HT application of other SAXS parameters (Porod coefficient (*P*_x_) and volume of correction (*V*_c_))^42,43^ and data transformations^44^ may offer further opportunities to kinetically monitor and refine SAR from SAXS screens.

Our exemplary pipeline for the redox-responsive flavoenzyme AIF is adaptable to other redox-responsive systems, including proteins containing regulatory redox-active disulfides^18^, metalloregulatory proteins^19^ and redox-sensitive photosensors such as cryptochromes^20^. Radio-caged ligands, currently developed as vehicles for X-ray-induced drug release in cancer treatment^45^, also offer the potential to enable HT-TR monitoring of targets lacking redox centers. Photocaged peptides and ligands are widely developed^46^ and commonly applied in TR-SAXS to stimulate biomolecular transitions^47^. Translating these tools to a radio-caged platform would allow coscreening of apo reference states and activated states stimulated by X-ray-triggered release of regulatory ions, substrates and peptides.

TR-SAXS is particularly suited to monitoring the evolution of biomolecular systems with challenging conformational heterogeneity, which are often inaccessible to conventional structural methods. Thus, future applications of this pipeline could also unlock drug discovery for conformationally linked kinetic processes, such as the disease-related formation of fibrils^48,49^ and multivalent biomolecular condensates^50^. Screening such kinetic mechanisms would require customizing experimental design with an initiatory stimulus (for example, uncaging nucleating peptides/small molecules or introducing temperature jumps). Similarly, kinetic evaluation of multistate targets existing as conformational ensembles (IDPs, modular proteins and nucleic acids) or conventional protein targets with flexible binding sites may triage compounds in ways opaque to endpoint binding and structural assays. As such, conformational kinetic screening has the potential to not only extend the frontiers of druggable targets but also contribute fundamental knowledge of biomolecular mechanisms and their related chemical biology.

This TR-HT-SAXS implementation is aided by the ability to translate experimental conditions from binding assays directly to HT-SAXS batch sample plates. As reported, our TR-HT-SAXS screening pipeline readily accommodates a 1–2% hit rate from the smaller, diverse chemical libraries (3,000–5,000 compounds) effective in academic applications of fragment and/or drug screening within a standard synchrotron shift^7,29^. Coordinating with beamline staff, users can expand this HT sample format to 100–200 fragments and multiplex further with fragment pooling. As such, this technology can enable academic labs to develop structure-based chemical probes targeting conformation, assembly and activity that complement and nuance genetic mutational and knockout approaches.

Increased signal and resolution in X-ray scattering measurements offer the potential to detect even smaller local conformational changes induced by fragment binding. In the current pipeline, averaging scattering data of replicate samples can increase signal to noise, extending the usable *q*-range to higher resolutions. The ongoing development of machine learning methods to denoise X-ray images may also enable digital noise reduction in the future^51^. Wide-angle X-ray scattering, which measures scattering at higher *q*-values, remains open to exploration for probing ligand-induced changes during drug discovery and may further expand the granularity and utility of SAXS ligand screens. Overall, many options exist for expanding and customizing TR-HT-SAXS conformational chemical discovery to a variety of contexts and targets. We anticipate that continued creation and analysis of HT multidimensional SAXS datasets will provide further opportunities to link chemistry, kinetics and macromolecular conformation.

## Methods

### Reagents

SYPRO Orange protein gel stain (5,000×) was obtained from Thermo Fisher Scientific (Life Technologies). Reduced β-NADH was purchased from Sigma-Aldrich (N0632). The following GL2500 library compounds were purchased from Life Chemicals: CX1 aminoquinolines 6-fluoroquinolin-4-amine (**C12**, ID: F2156-0068), 6-chloroquinolin-4-amine (**D1**, ID: F2156-0057) and 7-chloroquinolin-4-amine (**D3**, ID: F9995-2431) and reference scaffold quinolin-4-amine (**4AQ**, ID: F2179-0001). Atto488-NHS ester amine-reactive dye was obtained from Sigma-Aldrich (41698-1MG-F). BS^3^ (A39266) or disuccinimidyl suberate (A39267) amine cross-linker was acquired from Thermo Fisher Scientific.

### Plasmid construct and protein preparation

Wild-type AIF (78–613), AIF(W196A) (78–613) and AIF(H454A) (78–613) proteins were expressed and purified as previously described^21^. AIF(F482A) was subcloned into the wild-type AIF (78–613) background with Gibson cloning and expressed and purified as previously described^21^. Full-length CHCHD4 (1–142) isoform 1 (UniProt Q8N4Q1) was purchased from Integrated DNA Technologies as a G-block and subcloned with AgeI/Xho1 restriction sites into a customized pET30a vector with an N-terminal GB1-6×His fusion tag and Prescission protease cleavage site (a gift from the Henzler-Wildman laboratory while at Washington University School of Medicine in St Louis). This construct exhibited signs of read-through translation and was subsequently modified by Gibson assembly to introduce a second terminating stop codon. G-block and primer sequences are provided in Supplementary Data 1. CHCHD4 was expressed in Rosetta-gami 2(DE3) bacterial cells grown at 37 °C in shaker flasks and induced at an optical density at 600 nm (OD_600_) of ~0.6 with 1 mM IPTG for 3 h. Harvested bacterial pellets were stored at –20 °C until purification.

For CHCHD4 purification, thawed bacterial pellets were resuspended in lysis buffer (25 mM HEPES (pH 8), 150 mM NaCl, 10–30 mM imidazole, 1 mM PMSF and 0.1% Tween-20), homogenized with a glass pestle and sonicated to complete lysis (Qsonica). The lysate was clarified by centrifugation in a Beckman-Coulter JA25.50 rotor at 18,000 rpm (26,581*g*) for 20–30 min, filtered and loaded onto a 5-ml HisTrap column (GE Healthcare/Cytiva) using an Aktä Pure FPLC system. The column was washed with 10 column volumes of Ni-NTA Buffer A (25 mM HEPES (pH 8), 150 mM NaCl and 30 mM imidazole) and eluted with a 10-column volume gradient with Ni-NTA Buffer B (Ni-NTA Buffer A with 300 mM imidazole). Protein-containing fractions were pooled, treated with 6×His-tagged Prescission protease to remove fusion tags and exchanged overnight into dialysis buffer (25 mM HEPES (pH 7.5), 200 mM NaCl and 5 mM β-mercaptoethanol) at 4 °C. Dialysates were loaded onto a subtractive HisTrap column to separate cleaved fusion tags and free protein. The flow-through containing separated protein was concentrated to <1 ml and loaded onto a Superdex 75 10/300 column equilibrated in 25 mM HEPES (pH 7.5), 150 mM NaCl and 5 mM β-mercaptoethanol. Protein-containing fractions were pooled, concentrated, combined with 5% (vol/vol) glycerol and flash-frozen in liquid nitrogen for storage at –80 °C. Exemplary SDS–PAGE gels of wild-type AIF, AIF(W196A) and CHCHD4 size-exclusion chromatography purifications can be found in Supplementary Fig. 12.

### GL2500 fragment library

The customized in-house GL2500 fragment library is the original compound collection underlying the complete GLAD library (6,874 compounds) described by Moiani and colleagues^29^. GL2500 compounds were selected from the following fragment libraries offered by Life Chemicals: brominated (19%), fluorinated (19%), protein–protein interaction disruptors (15%), Fsp3 enriched (fragments containing sp3 hybridized carbon units; 19%) and Superior (fragments designed for solubility, low toxicity and cell permeability; 27%). Fragments were chosen for chemical diversity, absence of PAINS^52^ liabilities and predicted favorable physicochemical properties. The library was predispensed into deep 96-well blocks at 10 mg ml^–1^ (15–30 mM) in deuterated DMSO and stored at –30 °C.

### High-throughput differential scanning fluorescence screen

Purified AIF (78–613) and the GL2500 library were assembled into 384-well PCR plates for HT-DSF screening using a Beckman Biomek FX liquid-handling system as follows. Ten microliters of a 2× AIF (1 mg ml^–1^) and SYPRO Orange dye (1:1,000 dilution) stock was dispensed into target PCR plates. Plates were spun briefly to bring contents to the bottom of the well. Screening compounds were prediluted at a 1:10 ratio into screening buffer (25 mM HEPES (pH 7.5) and 150 mM NaCl), and 10 µl of diluted compound was transferred and mixed into the protein–dye solution. Plates were centrifuged a final time and sealed for DSF acquisition. Each 384-well plate contained three 96-well library plates and a fourth quadrant dedicated to replicate control samples (buffer, DMSO (5%), NADH (83 µM, tenfold molar excess) and combined DMSO/NADH). Final AIF protein and screening compound concentrations were 8.3 µM (0.5 mg ml^–1^) and 0.75–1.5 mM (0.5 mg ml^–1^, ~90- to 180-fold molar excess), respectively.

DSF melt curves were measured on an Applied Biosystems QuantStudio 6 Flex Real-Time PCR instrument with reads every 1–2 °C across a 25–99 °C temperature ramp. *T*_m_ values were calculated by Boltzmann fitting of the melt curves in GraphPad Prism 9.0. Melt curves exhibiting fluorescent interference or signs of sample aggregation were excluded from further analysis (~23% of all samples). Plate-to-plate variability among AIF–DMSO controls was low (coefficient of variation of <1%; Extended Data Fig. 1a), allowing a screen-wide reference *T*_m_ (65.6 °C) and standard deviation (*σ* = 0.6 °C) to be defined (*N* = 216). Using AIF–DMSO *T*_m_ variation *σ* as an empirical estimate of screen-wide Δ*T*_m_ uncertainty, we defined a hit cutoff of 3 s.d. from the AIF–DMSO *T*_m_ mean (| Δ*T*_m_ | > 1.7 °C). Qualifying fragments were collated and ranked, resulting in 19 *T*_m_-elevating compounds (1.0%) and 46 *T*_m_-lowering compounds (2.4%). Compounds lowering *T*_m_ were included because the AIF native NADH ligand lowers AIF *T*_m_ ~11 °C. Of the 46 *T*_m_-lowering hits, the top 20, which were closest in impact to the native NADH ligand, were selected for further characterization and assembled with *T*_m_-elevating fragments into a 39-compound sublibrary for verification (Supplementary Table 2).

HT-DSF screening of 2,500 fragments was completed within 1 week and could reasonably accommodate 3,000–5,000 compounds within 7–10 days. Further details are reported in Supplementary Table 1. Fragment hits were dispensed into a separate 96-well plate for follow-up and assigned a reference ID based on their well position (B1–E3).

### Differential scanning fluorescence verification screen

The DSF verification screen was assembled similar to the HT version. A 2× stock solution of purified AIF (78–613) (1 mg ml^–1^) and SYPRO Orange dye (1:1,000) were prepared in screening buffer. Hit fragments were prediluted in screening buffer (1 µl of GL2500 compound + 18 µl of screening buffer), combined with 20 µl of 2× AIF/SYPRO Orange stock and loaded into 96-well PCR plates. DSF thermal melts were measured with a Bio-Rad CFX Connect Real-Time PCR System and analyzed with CFX Maestro software (version 1.0). A reference *T*_m_ (62.0 °C) and standard deviation (*σ* = 0.2 °C) from AIF–DMSO controls (*N* = 6) were calculated and used to estimate uncertainty within the verification screen (Extended Data Fig. 1c). Twenty-seven of the 39 HT-DSF hits exhibited *T*_m_ deviations greater than 3 s.d. (| Δ*T*_m_ | > 0.7 °C) relative to DMSO controls, with the highest hit reproducibility occurring among *T*_m_-lowering compounds (Extended Data Fig. 1b).

### Microscale thermophoresis verification screen

Atto488-labeled AIF (78–613) was prepared by incubating 2 mg ml^–1^ purified protein with a two to three molar excess of Atto488-NHS ester (Sigma-Aldrich, 41698) in reaction buffer (equal parts 25 mM HEPES (pH 7.5), 150 mM NaCl and 0.2 M sodium bicarbonate, pH 9) in the dark for 1 h at room temperature. Free label was removed by desalting the reaction on a PD-10 gravity flow column (GE Healthcare/Cytiva) equilibrated in screening buffer. Purified Atto488-AIF was checked for degree of labeling by UV-Vis spectroscopy, aliquoted and flash-frozen in liquid nitrogen for storage at –80 °C.

MST reactions were assembled by prediluting 1 µl of 10 mg ml^–1^ GL2500 hit fragments or DMSO in 14 µl of MST buffer (25 mM HEPES (pH 7.5), 150 mM NaCl and 0.01% Tween-20), adding 15 µl of 2× Atto488-AIF (78–613) (200 nM) and incubating at room temperature for 15 min in the dark. Reactions were loaded into standard silica capillaries, and MST measurements were acquired on a Monolith NT.115 system (NanoTemper, PR.ThermControl 2.1.6) at 25 °C with 30% LED power and 40% infrared excitation for 20 s with 3-s equilibration and 1-s recovery periods. Time-averaged amplitudes were calculated over a 1-s window (4 to 5 s) during excitation, and three consecutive scans were averaged to generate final values. MST amplitudes were determined using NanoTemper PR.Stability Analysis 1.0.2 and exported to Microsoft Excel (version 2303) for analysis. A reference response amplitude (956.3 response units) and standard deviation (*σ* = 1.4) were defined from AIF–DMSO controls (*N* = 4) and used to estimate uncertainty within the MST screen (Extended Data Fig. 1c). Seventeen of the 39 HT-DSF hits exhibited MST response amplitudes exceeding 3 s.d. (response units > 5.2).

### TR-HT-SAXS conformational and kinetic screening and analysis

Purified AIF (78–613) (300 µl, 60 mg ml^–1^) was exchanged into screening buffer (25 mM HEPES (pH 7.5) and 150 mM NaCl) by gel filtration chromatography on a Superdex 200 Increase 10/300 GL column at 0.3 ml min^–1^ with collection of 0.5-ml fractions. Fractions from the eluted peak were pooled and diluted to a 4.1 mg ml^–1^ protein stock; buffer fractions from the void volume were pooled to prepare protein dilutions and matched sample blanks. SAXS samples were assembled by combining 1 µl of 10 mg ml^–1^ GL2500 compound with 29 µl of AIF protein stock. Duplicate samples for buffer subtraction were prepared by combining 2 µl of GL2500 compound with 58 µl of buffer. Final AIF and fragment concentrations were ~67 µM and 0.5–1.0 mM for ~8- to 16-fold molar excess of ligand. Control samples with buffer, DMSO, NADH (20 mM stock for 600 µM or tenfold molar excess final concentration) and DMSO/NADH were prepared in triplicate. Samples (25 µl) were transferred into two 96-well PCR plates, sealed and flash-frozen with liquid nitrogen for shipment on dry ice to the SIBYLS SAXS beamline (12.3.1)^9,10,12^ at the Advanced Light Source at Lawrence Berkeley National Laboratory. Two additional SAXS experiments of WT AIF combined with DMSO solutions of CX1 compounds, prepared from purchased commercial powders, were collected at separate times to verify the time-dependent stimulation of AIF dimerization. SAXS samples for purified AIF(W196A) (78–613) and CX1 chemotype fragments were prepared in a similar manner from 50 mM CX1 DMSO stocks prepared from commercial powder. Final sample concentrations were 4 mg ml^–1^ AIF(W196A) and 1.7 mM compound.

TR-SAXS data sets were collected in 0.3-s exposures (WT AIF/GL2500 hits) or 0.2-s exposures (AIF(W196A)/CX1 fragments) for 10 s at an X-ray wavelength of 1.27 Å and 1.5-m sample-to-detector distance, corresponding to a scattering vector *q*-range of 0.01 to 0.59 Å^–1^ (*q* = 4*π* × sin(*θ*)/*λ*, where 2*θ* is the scattering angle), with a Pilatus3 2M pixel array detector. Measurement of the 39-fragment TR-HT-SAXS screen was completed within an 8-h synchrotron shift; screens of 100–200 compounds can be reasonably accommodated by one to two synchrotron shifts^7^. All samples were successfully acquired apart from nonverified fragment **C5**, which was disrupted by a bubble in the sample cell, and one AIF–DMSO control, which exhibited poor background subtraction. X-ray scattering datasets were reduced and integrated using automated in-house scripts from SIBYLS. Duplicate buffer-matched datasets were processed similarly and were averaged and subtracted as time-matched exposures from sample data to yield TR-SAXS series for each sample. Scattering curves were inspected for signs of radiation damage and aggregation by Guinier analysis using Primus 3.0.1. *I*(0) and *R*_g_ values were derived using ScÅtter 3.0 (https://bl1231.als.lbl.gov/scatter/) and exported to Excel and GraphPad Prism for further analysis and graphical display. Standard deviations for *I*(0) and *R*_g_ were calculated from two (AIF–DMSO) or three (AIF–NADH–DMSO) controls.

*V*_R_ similarity values and SSM maps^32^ were calculated and clustered with the SAXS similarity web application hosted by the SIBYLS website (https://sibyls.als.lbl.gov/saxs-similarity/) using a *q-*range of 0.015–0.15 Å^–1^. This application uses a single-linkage AHC algorithm to sort Euclidean distances between paired *V*_R_ values. To verify the robustness and consistency of chemotype clusters identified by the AHC approach, each time point was also independently clustered using a *k*-means approach with the ‘NbClust’ function^53^ in R (version 4.3.1)^54^. Optimal cluster number at each time point was determined in ‘NbClust’ by maximizing the Krzanowski–Lai index^55^. For *t* = 2.7 s, the Krzanowski–Lai index selected an off-trend two clusters. Thus, the 2.7-s clustering is also displayed with results obtained from the ‘kmeans’ function from R’s Cluster package with four clusters specified (Extended Data Fig. 5c), which more accurately reflects the time series trend.

Estimates of *V*_R_ uncertainty were generated in Excel from time-resolved *V*_R_ values generated from AIF–DMSO and AIF–NADH–DMSO controls (monomer–dimer *V*_R_ values and dimer–dimer self-*V*_R_ values) as follows. *V*_R_ standard deviations were calculated from two independent replicates at each time point and averaged across each time series to define a standard uncertainty. A *V*_R_ significance threshold was then calculated as three times the averaged standard uncertainty beyond the *V*_R_ average at each time point (Fig. 2a, dotted line).

Kinetic analysis of *V*_R_ values was performed in GraphPad Prism. First-order rate constants, *k*_VR_, were calculated by fitting each *V*_R_ time series to a single decaying exponential. CX1 and CX2 *k*_VR_ values were clustered with single-linkage AHC and *k*-means algorithms using the ‘hclust’ and ‘kmeans’ functions on Euclidean distances of paired *k*_VR_ values in R^54^. Minimization of the total within-cluster sum of squares (elbow method) was used to select optimal *k*-means cluster number.

### Amine cross-linking experiments

For the CX1 chemotype, reactions (10 µl) of wild-type AIF (10 µM) with 5% DMSO, 100 µM NADH or 5 mM CX1 ligands (**C12**, **D1** and **D3**) were prepared in screening buffer and incubated for 10 min at room temperature. For wild-type AIF, AIF(F482A) and AIF(H454A) mutant assessment, 10 µM protein was combined with buffer or 100 µM NADH and incubated similarly. Binding reactions were combined with a final concentration of 1.25 mM BS^3^ or disuccinimidyl suberate amine cross-linker and incubated for an additional 30 min before quenching with SDS loading buffer. Cross-linked species were visualized by gradient (4–20%) SDS–PAGE. Displayed gels are representative of three independent experiments.

### UV-Vis absorbance monitoring of AIF CTC formation

Absorbance spectra of wild-type and mutant AIF were measured for the UV-Vis wavelength range (200–800 nm) using a Cary 60 UV-Vis spectrophotometer (Agilent Technologies, Scanview application version 5.1.0.1016 (firmware version 6.4.0.141; hardware version 2.00)). Protein samples were prepared at 20 µM, combined with 100 µM NADH and scanned every 2.5 min for 15 min. FAD reduction was monitored as a loss of absorbance at 450 nm and FADH^–^/NAD^+^ CTC formation as a rise in absorbance at 600–800 nm for two independent experiments.

### AIF MST titrations with CX1 ligands

Atto488-labeled AIF(W196A) (78–613) was prepared as described above for the wild-type protein. CX1 chemotype ligands were titrated into Atto488-AIF or Atto488-AIF(W196A) protein (100 nM) following a 16-point, twofold dilution series from 150 nM to 5 mM compound. Reactions were incubated in the dark for 15 min at room temperature and loaded into standard silica capillaries. MST measurements were acquired as described above. Binding curves were normalized with NanoTemper’s MO.Affinity Analysis software and fit to a one-site binding model using GraphPad Prism software.

### CHCHD4 MST titrations with AIF in the presence of CX1 ligands

Atto488-labeled CHCHD4 was prepared as described above. For these experiments, MST binding buffer was supplemented with 5 mM TCEP. Wild-type AIF or AIF(W196A) mutant protein was titrated from 0.9 nM to 30 µM into 100 nM Atto488-CHCHD4 combined with 1.25 mM CX1 ligands, 2.5% DMSO or 2.5% DMSO with 300 µM NADH. Reactions were incubated in the dark for 15 min at room temperature and loaded into standard silica capillaries. MST measurements were acquired and analyzed as described above.

### Crystallization and structure determination of AIF–aminoquinoline complexes

Crystals of wild-type AIF (78–613) were grown by hanging-drop vapor diffusion (1 µl of protein:1 µl of reservoir solution) with protein solutions of 10–12 mg ml^–1^ in 0.1 M Tris (pH 8.5), 0.34–0.38 M Li_2_SO_4_ and 25–26% (wt/vol) PEG3350 at 22 °C. For compound soaks, crystals of wild-type AIF were transferred to 2-µl drops containing soak solutions in low-profile 96-well plates (50-µl reservoir), sealed and incubated for 1–2 h at 22 °C. Soak solutions contained 0.1 M Tris (pH 8.5), 0.4 M Li_2_SO_4_ and 25% (wt/vol) PEG3350 supplemented with 5% (vol/vol) DMSO and ~2.8 mM 6-fluoro-2-methylquinolin-4-amine (**C9**; Life Chemicals, F2156-0070), 8-fluoro-2-methylquinolin-4-amine (**C11**; Life Chemicals, F2156-0047) or 8-methoxyquinolin-4-amine (**D7**; Life Chemicals, F2183-0014). Crystals were briefly exchanged into cryoprotectant buffer (15% (vol/vol) ethylene glycol – 8.5 µl soak reservoir + 1.5 µl ethylene glycol) and flash-cooled in liquid nitrogen.

Wild-type AIF was cocrystallized with 7-chloroquinolin-4-amine (**D3**; Life Chemicals, F9995-2431) in 0.1 M Tris (pH 8.5), 0.38 M Li_2_SO_4_ and 21% (wt/vol) PEG3350 using 10 mg ml^–1^ AIF protein solution mixed with 1 mM compound. AIF–D3 crystals were collected without additional soaking, briefly exchanged into cryoprotectant buffer (15% (vol/vol) ethylene glycol – 8.5 µl of 0.1 M Tris (pH 8.5), 0.4 M Li_2_SO_4_, 25% (wt/vol) PEG3350 and 1 mM **D3** + 1.5 µl of ethylene glycol) and flash-cooled in liquid nitrogen.

Crystals of AIF(W196A) were grown by hanging-drop vapor diffusion, as described above, in 0.1 M HEPES (pH 7.0–8.5), 0.30–0.32 M Na_2_SO_4_ and 16–20% (wt/vol) PEG3350. Crystals were soaked as described earlier using 0.1 M HEPES (pH 7.0–8.5), 0.30–0.32 M Na_2_SO_4_ and 16–20% (wt/vol) PEG3350 supplemented with 17% (vol/vol) DMSO and 10 mM quinolin-4-amine (**4AQ**; Life Chemicals, F2179-0001) or 0.1 M Tris (pH 8.5), 0.35 M NaCl and 25% (wt/vol) PEG3350 supplemented with 10% DMSO (vol/vol) and 5 mM 6-fluoroquinolin-4-amine (**C12**; Life Chemicals, F2156-0068), 6-chloroquinolin-4-amine (**D1**; Life Chemicals, F2156-0057) or 7-chloroquinolin-4-amine (**D3**; Life Chemicals, F9995-2431). Crystals were briefly exchanged into cryoprotectant buffer (15% (vol/vol) ethylene glycol – 8.5 µl of soak reservoir + 1.5 µl of ethylene glycol) and flash-cooled in liquid nitrogen.

Synchrotron X-ray diffraction data from wild-type AIF crystals were collected at the Advanced Light Source beamline 8.3.1 at Lawrence Berkeley National Laboratory with cryostream (100 K) at an X-ray wavelength of 1.1158 Å. X-ray data from AIF(W196A) crystals were collected at the FMX beamline (17-ID-2) at the National Synchrotron Light Source-II at Brookhaven National Laboratory^56^ under cryostream (100 K) at an X-ray wavelength of 0.9793 Å. X-ray data were processed with fast_dp^57^ (FMX) or XDS (version March 2019)^58^ and CCP4 (version 7.0)^59^ (BL 8.3.1). Structures were solved by molecular replacement with the Phaser^60^ module in Phenix 1.18.2^61^ using coordinates PDB 4BV6 (wild-type AIF) or PDB 5KVH (AIF(W196A)). Structures were refined with Phenix^62^, and model building was performed in Coot (v. 0.8.9)^63^. Polder maps^64^ of GL2500 ligands and composite omit maps with simulated annealing^65^ for allosteric residues in the AIF active site were calculated with Phenix. Favored and allowed Ramachandran populations for refined AIF structures were as follows: AIF(W196A)–**C12** (97.3%/2.7%), AIF(W196A)–**D1** (97.3%/2.7%), AIF(W196A)–**D3** (96.6%/3.4%), AIF(W196A)–**4AQ** (97.1%/2.9%), AIF–**D3** (96.8%/3.2%), AIF–**C9** (97.0%/3.0%), AIF–**C11** (96.6%/3.4%) and AIF–**D7** (96.6%/3.4%). Structural coordinates and crystallographic structure factors have been deposited with the PDB. Molecular visualization and analysis were performed with UCSF Chimera (version 1.14)^66^. Crystallographic and molecular visualization software were also accessed through the SBGrid^67^.

### Reporting summary

Further information on research design is available in the Nature Portfolio Reporting Summary linked to this article.

## Online content

Any methods, additional references, Nature Portfolio reporting summaries, source data, extended data, supplementary information, acknowledgements, peer review information; details of author contributions and competing interests; and statements of data and code availability are available at 10.1038/s41589-024-01609-1.

### Supplementary information

Supplementary Information Supplementary Figs. 1–14, Tables 1–7 and references.

Reporting Summary

Supplementary Data 1 G-block and primer sequences.

Supplementary Data 2 Source data for Supplementary Fig. 1.

Supplementary Data 3 Source data for Supplementary Fig. 2.

Supplementary Data 4 Source data for Supplementary Fig. 3.

Supplementary Data 5 Source data for Supplementary Fig. 4.

Supplementary Data 6 Source data for Supplementary Fig. 5.

Supplementary Data 7 Source data for Supplementary Fig. 6.

Supplementary Data 8 Source data for Supplementary Fig. 7.

Supplementary Data 9 Source data for Supplementary Fig. 9.

### Source data

Source Data Fig. 1 Statistical source data for Fig. 1.

Source Data Fig. 2 Statistical source data for Fig. 2.

Source Data Fig. 3 Statistical source data for Fig. 3.

Source Data Fig. 4 Statistical source data for Fig. 4 and unprocessed gel for Fig. 4a.

Source Data Fig. 5 Statistical source data for Fig. 5 and unprocessed gels for Fig. 5d.

Source Data Extended Data Fig. 1 Statistical source data for Extended Data Fig. 1.

Source Data Extended Data Fig. 2 Statistical source data for Extended Data Fig. 2.

Source Data Extended Data Fig. 3 Statistical source data for Extended Data Fig. 3.

Source Data Extended Data Fig. 4 Statistical source data for Extended Data Fig. 4.

Source Data Extended Data Fig. 5 Statistical source data for Extended Data Fig. 5.

## Data Availability

Structural coordinates used for crystallographic molecular replacement calculations and AIF images (Fig. 1b) were accessed from the Protein Data Bank (PDB; https://www.rcsb.org/) from entries 4BV6 (wild-type AIF, monomer), 5KVH (AIF(W196A)) and 4BUR (AIF–NADH, dimer). Structural coordinates and crystallographic structure factors of AIF–aminoquinoline complexes have been deposited with the Protein Data Bank (https://www.rcsb.org/) as follows: AIF(W196A)–**C12** (PDB 8D3E), AIF(W196A)–**D1** (PDB 8D3G), AIF(W196A)–**D3** (PDB 8D3H), AIF(W196A)–**4AQ** (PDB 8D3I), AIF–**D3** (PDB 8D3N), AIF–**C9** (PDB 8D3J), AIF–**C11** (PDB 8D3K) and AIF–**D7** (PDB 8D3O). AIF–ligand SAXS datasets from 0.3-s (oxidized) and 2.1-s (reduced) exposures have been deposited with the SIMPLE SCATTERING repository^33^ (https://simplescattering.com/) under the code XS97QA1S. The authors declare that the data supporting the findings of this study are available within the paper and its associated Supplementary Information files. Should any raw data files be needed in another format, they are available from the corresponding author upon reasonable request. Source data are provided with this paper.
