# Supplementary material for: Chemical screening by time-resolved X-ray scattering to discover allosteric probes
Source: Nat Chem Biol. Author manuscript; Available in PMC 2024 Sep 1. (PMC11358040; doi:10.1038/s41589-024-01609-1)
Supplement: Supplementary Information [file NIHMS2003799-supplement-Supplementary_Information.pdf]

nature chemical biology

Article

https://doi.org/10.1038/s41589-024-01609-1

Chemical screening by time-resolved X-ray scattering to discover allosteric probes

In the format provided by the authors and unedited

Table of Contents

Supplementary Figures

Supplementary Figure 1. GL2500 time-resolved V_R SAXS similarity matrix (SSM) maps ... 3
Supplementary Figure 2. TR-SAXS characterization of the CX1 chemotype ... 4
Supplementary Figure 3. TR-SAXS characterization of the CX2 chemotype (C9-D7) ... 5
Supplementary Figure 4. TR-SAXS characterization of the CX2 chemotype (D8-E3) ... 6
Supplementary Figure 5. TR-SAXS characterization of the CX3 and CX4 chemotypes ... 7
Supplementary Figure 6. TR-SAX characterization of the CX4 chemotype (B8-C4) ... 8
Supplementary Figure 7. SAXS k_{VR} exponential fits for CX1 and CX2 chemotypes ... 9
Supplementary Figure 8. Counter SAR from 4-aminoquinolines excluded from GL2500 HT-DSF screening hits ... 10
Supplementary Figure 9. TR-SAXS characterization of AIF-W196A with CX1 chemotype ligands ... 11
Supplementary Figure 10. AIF-WT and AIF-W196A active site SA-omit maps ... 12
Supplementary Figure 11. Uncropped SDS-PAGE gels of wild-type and mutant AIF cross-linking reactions ... 13
Supplementary Figure 12. SDS-PAGE analysis of AIF-WT, AIF-W196A, and CHCHD4 SEC profiles ... 14
Supplementary Figure 13. Chemical 2D Diagrams of GL2500 Fragment Hits and 4-aminoquinoline scaffold ... 15
Supplementary Figure 14. Uncropped gel images from Supplementary Fig. 12 ... 16

Supplementary Tables

Supplementary Table 1. Small molecule screening data ... 17
Supplementary Table 2. AIF HT-DSF GL2500 fragment hits. ... 19
Supplementary Table 3. SAXS collection and analysis parameters ... 20
Supplementary Table 4. Wild-type and W196A AIF affinities for mitochondrial partner CHCHD4 stimulated by native NADH or CX1 chemotype ligands ... 22
Supplementary Table 5. AIF-GL2500 summary of crystallographic structures ... 22
Supplementary Table 6. X-ray data collection and refinement statistics for AIF-W196A-GL2500 complexes ... 23
Supplementary Table 7. X-ray collection and refinement statistics for AIF-WT-GL2500 complexes ... 24

Supplementary References ... 25

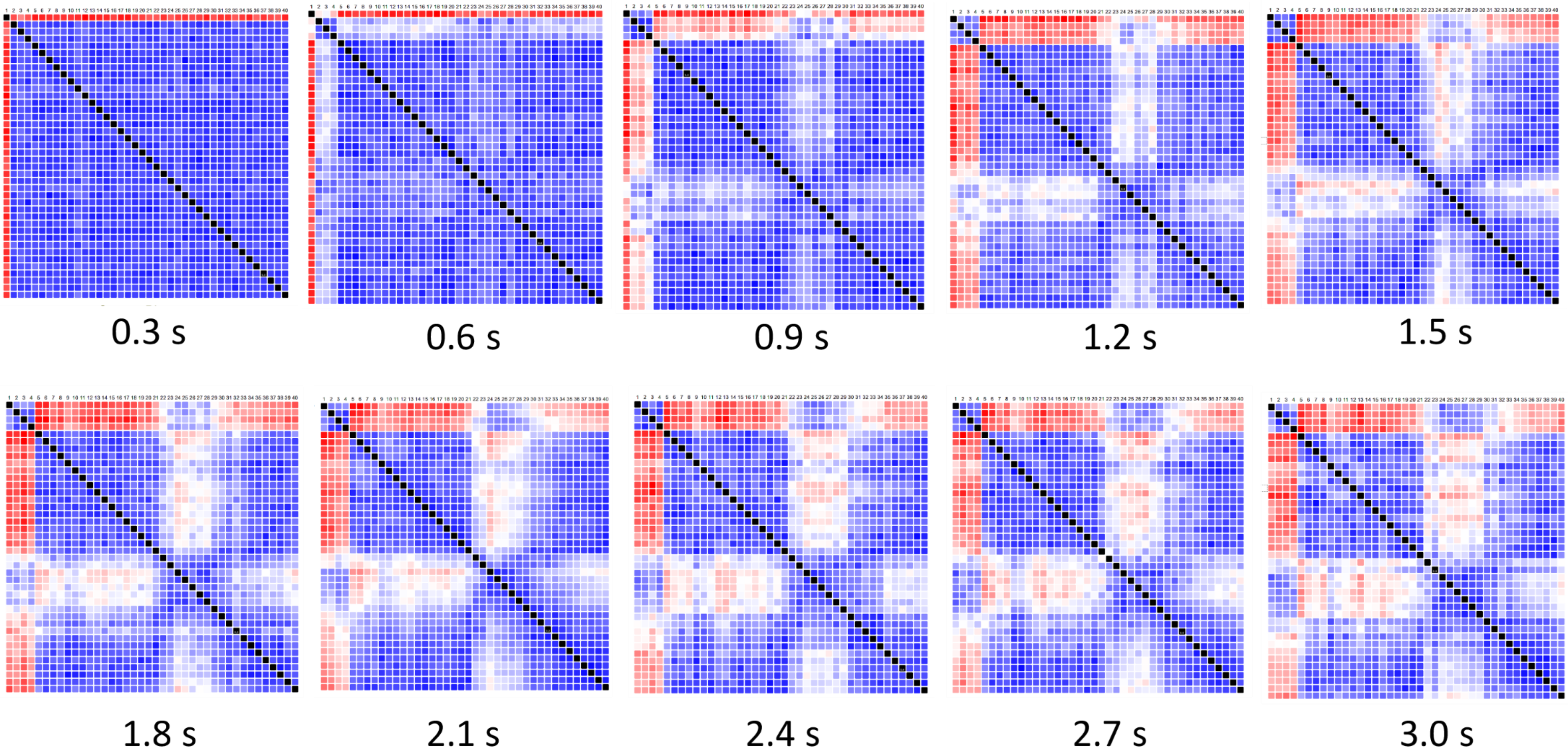

Supplementary Figure 1. GL2500 time-resolved V_R SAXS similarity matrix (SSM) maps. Samples within matrices are sorted using the clustered ranking from the SSM at 2.1 s.

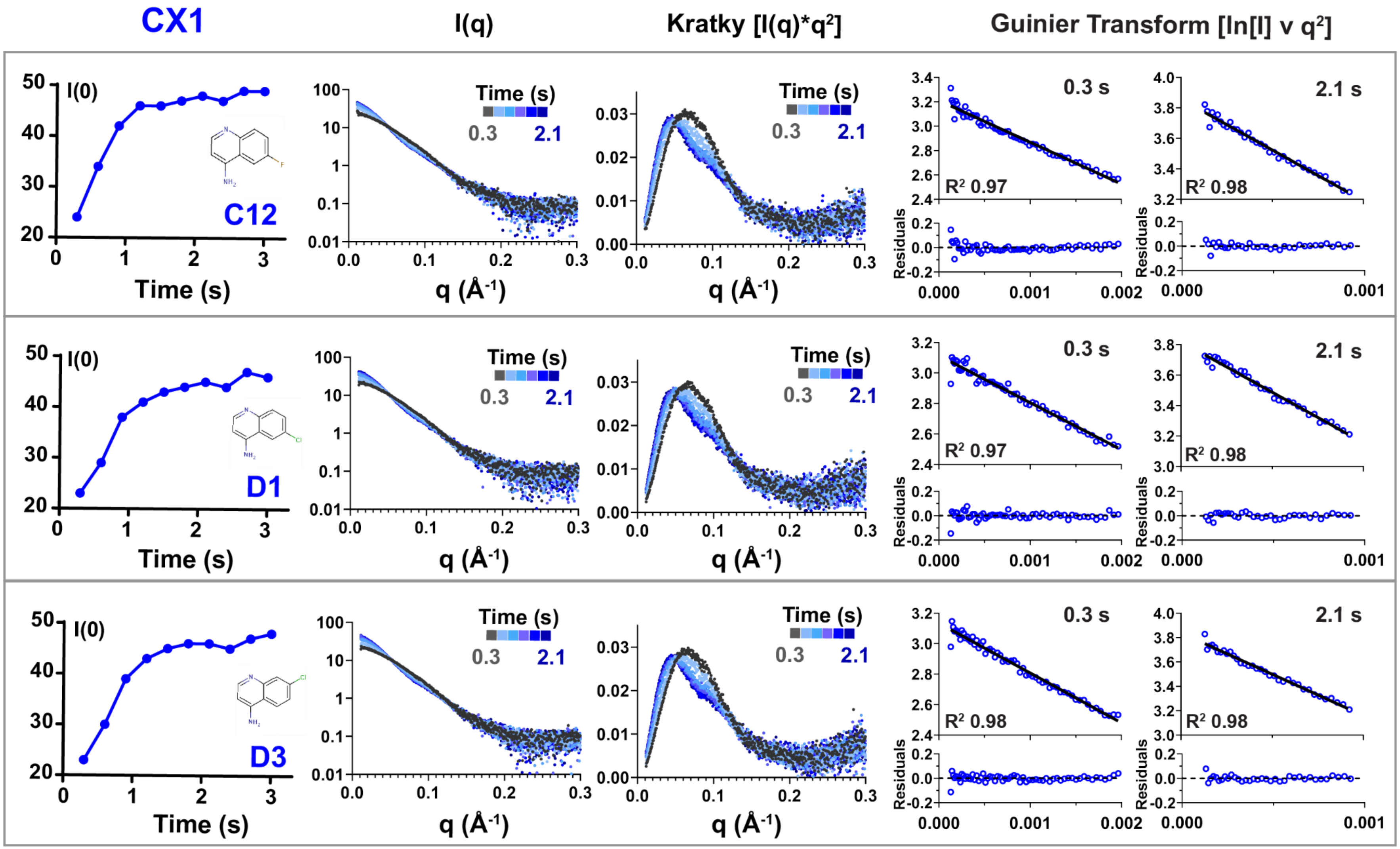

Supplementary Figure 2. TR-SAXS characterization of the CX1 chemotype. Overlays of I(q) and Kratky transforms at 0.3, 0.6, 1.2,1.5, 1.8, and 2.1 s. Linear Guinier transforms at initial and final exposures indicate absent aggregation.

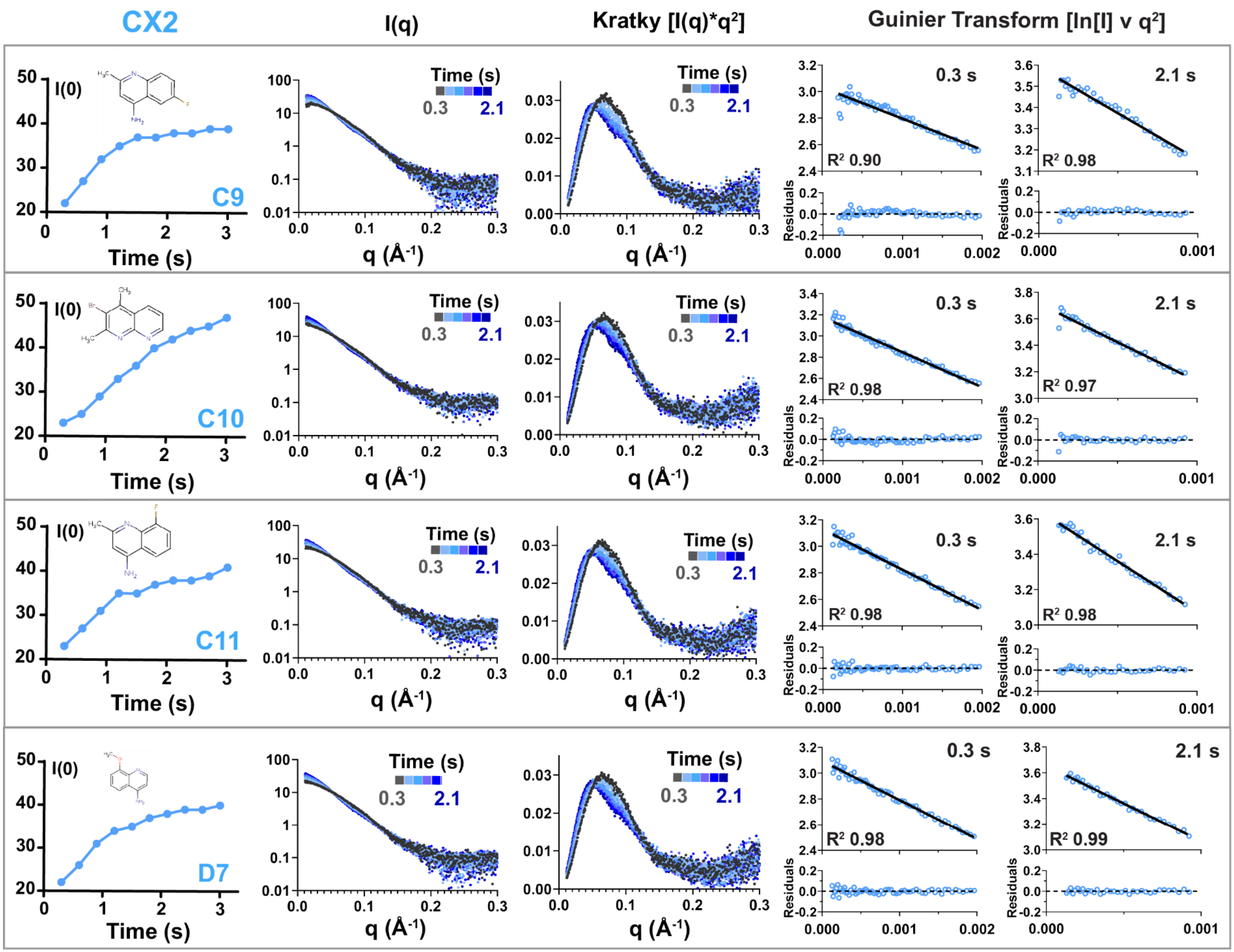

Supplementary Figure 3. TR-SAXS characterization of the CX2 chemotype (C9-D7). Overlays of I(q) and Kratky transforms at 0.3, 0.6, 1.2,1.5, 1.8, and 2.1 s. Linear Guinier transforms at initial and final exposures indicate absent aggregation.

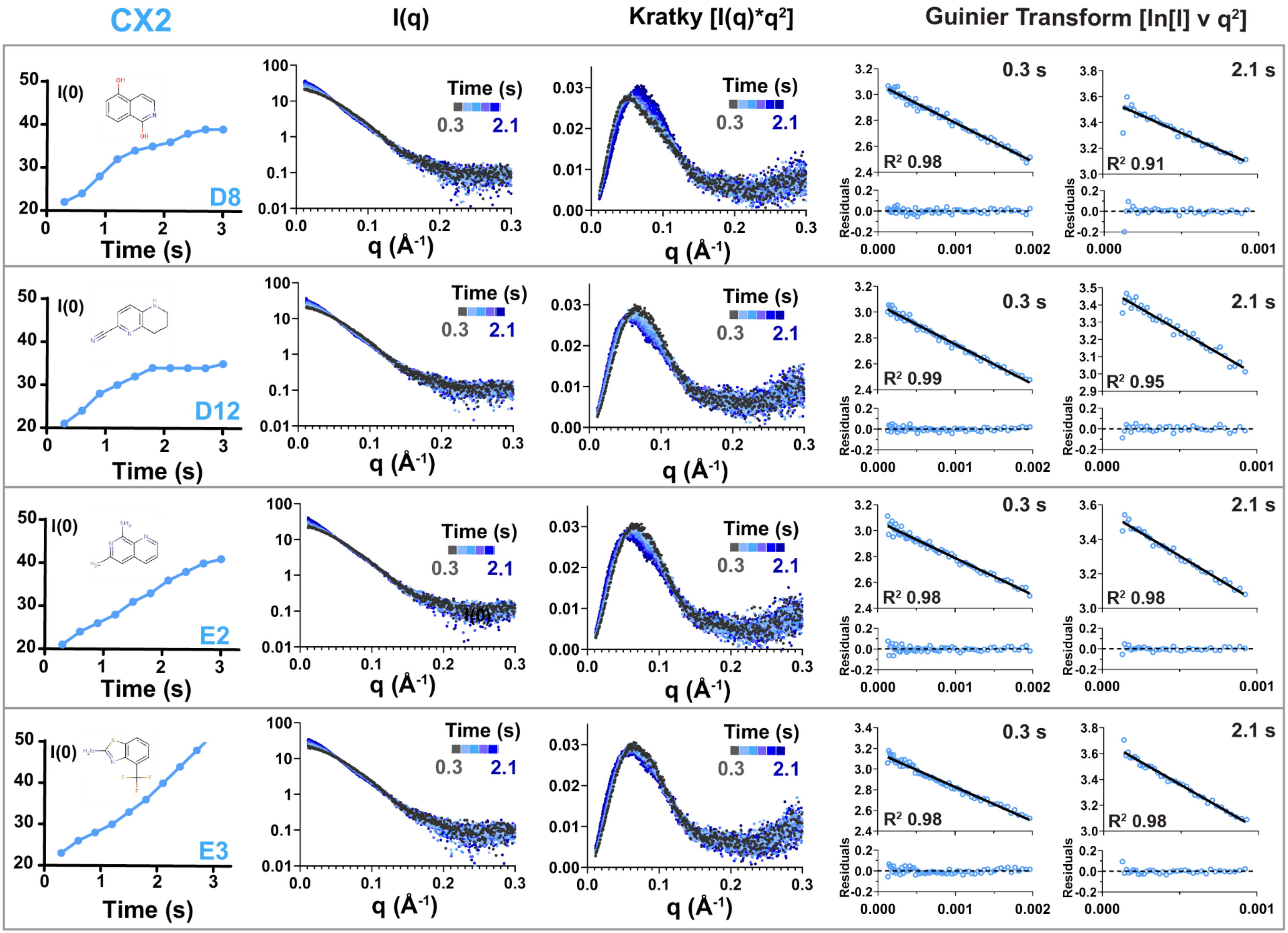

Supplementary Figure 4. TR-SAXS characterization of the CX2 chemotype (D8-E3). Overlays of I(q) and Kratky transforms at 0.3, 0.6, 1.2,1.5, 1.8, and 2.1 s. Linear Guinier transforms at initial and final exposures indicate absent aggregation.

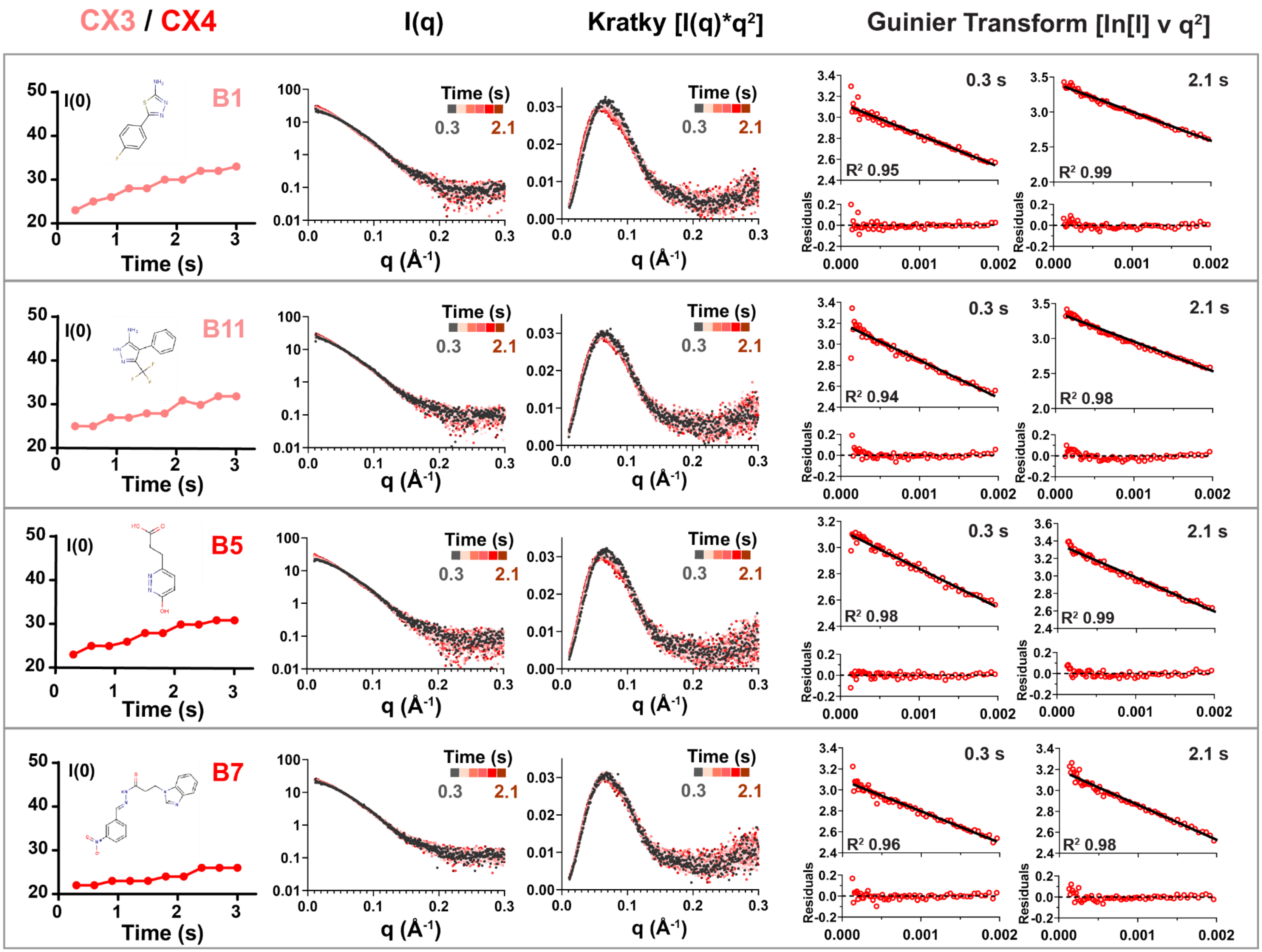

Supplementary Figure 5. TR-SAXS characterization of the CX3 and CX4 chemotypes. Overlays of I(q) and Kratky transforms at 0.3, 0.6, 1.2,1.5, 1.8, and 2.1 s. Linear Guinier transforms at initial and final exposures indicate absent aggregation.

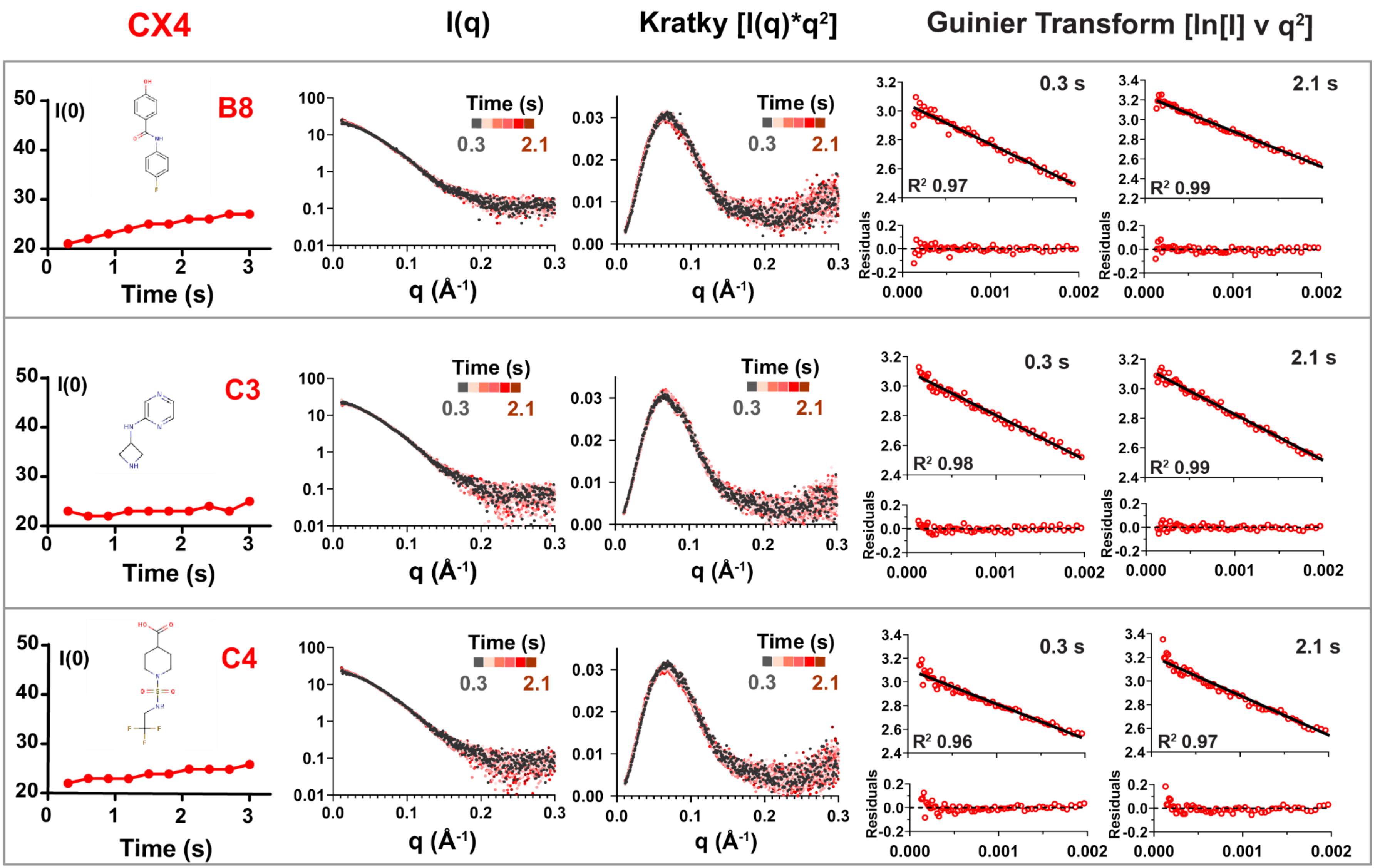

Supplementary Figure 6. TR-SAXS characterization of the CX4 chemotype (B8-C4). Overlays of I(q) and Kratky transforms at 0.3, 0.6, 1.2,1.5, 1.8, and 2.1 s. Linear Guinier transforms at initial and final exposures indicate absent aggregation.

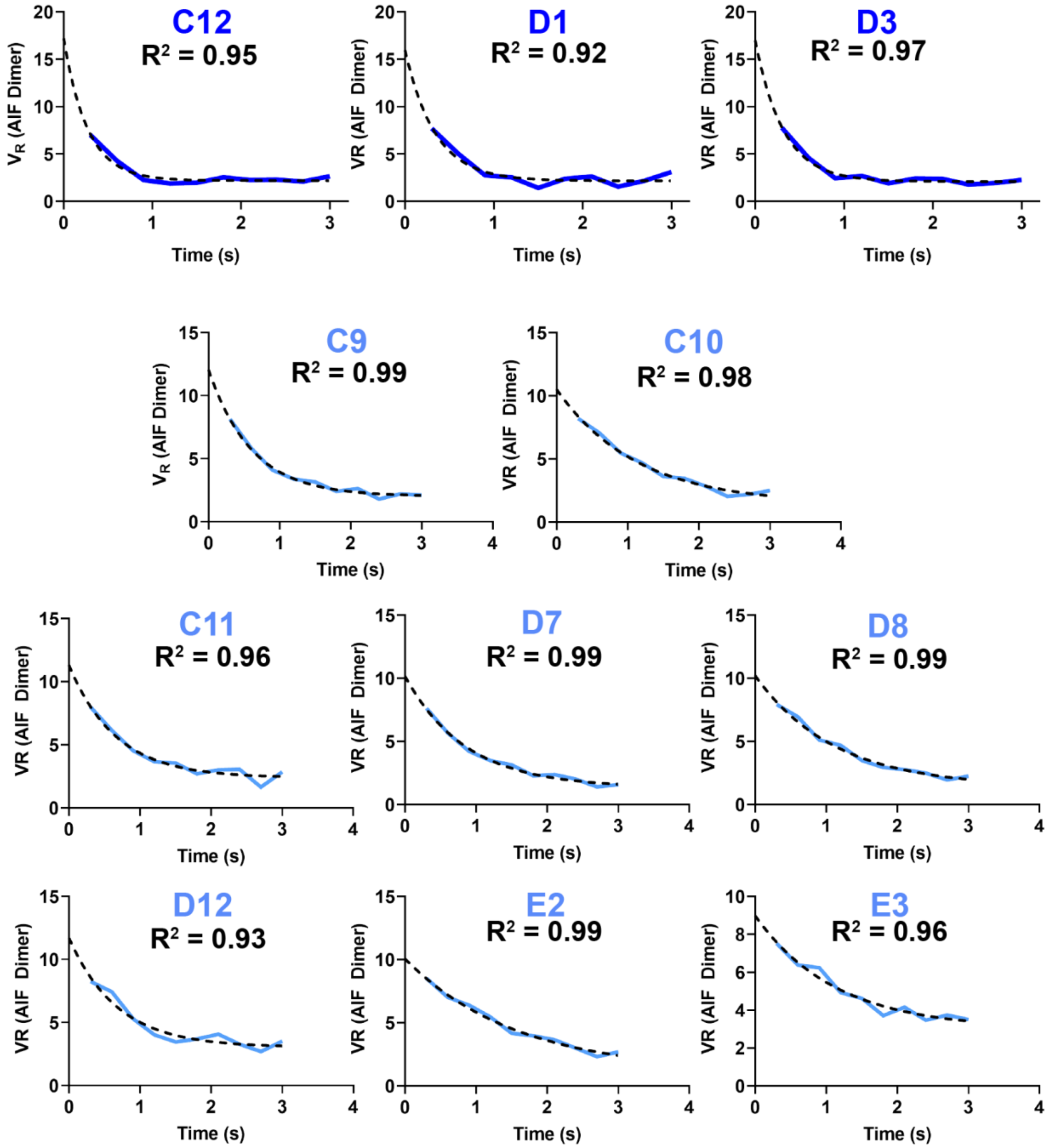

Supplementary Figure 7. SAXS k_{VR} exponential fits for CX1 and CX2 chemotypes.

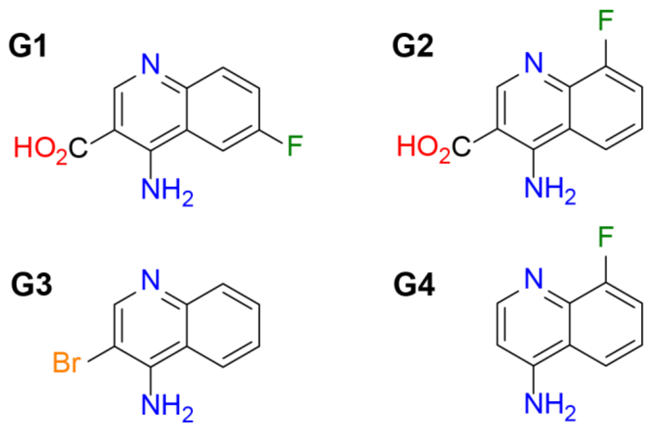

Supplementary Figure 8. Counter SAR from 4-aminoquinolines excluded from GL2500 HT-DSF screening hits.

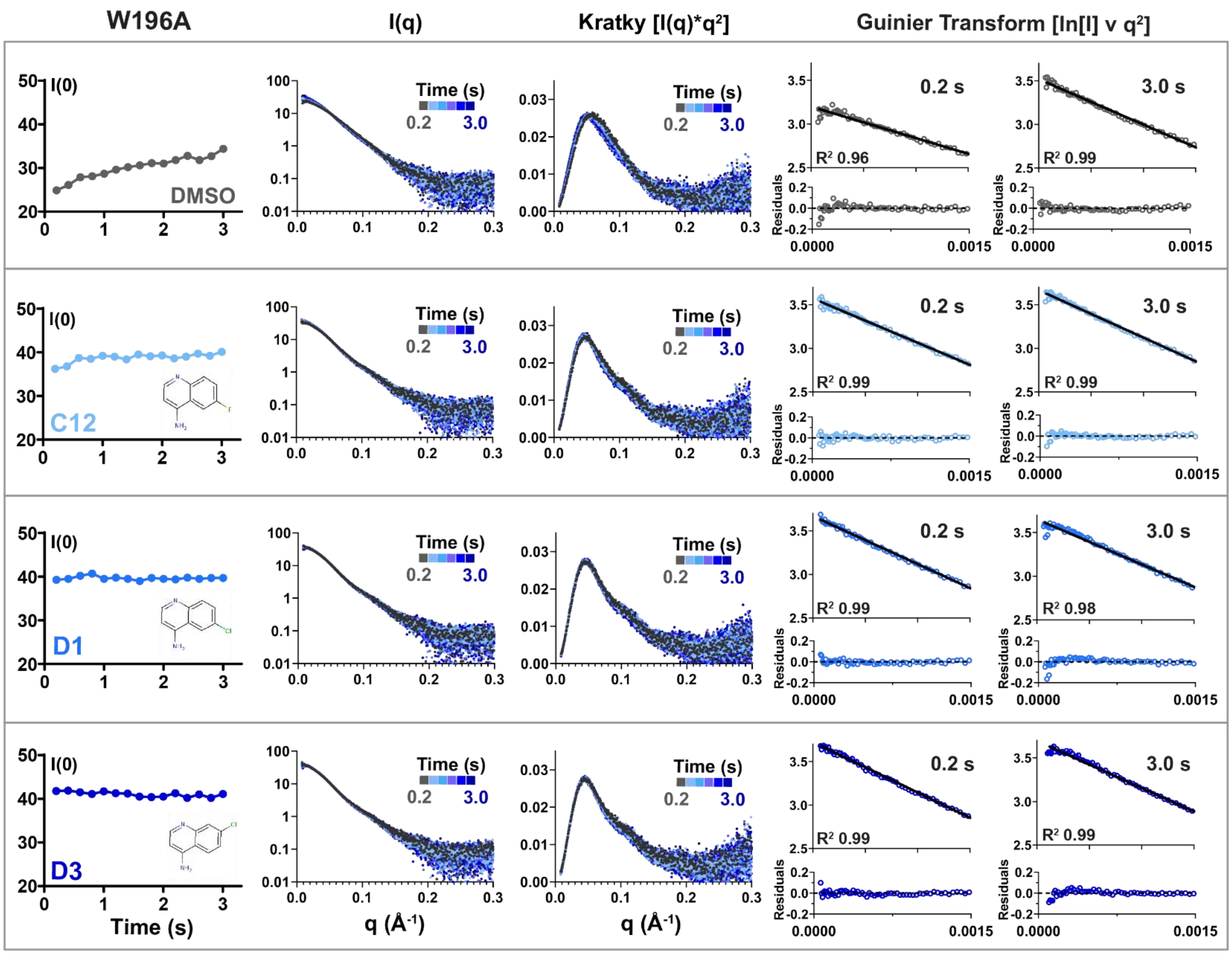

Supplementary Figure 9. TR-SAXS characterization W196A with CX1 chemotype fragments. Overlays of I(q) scattering curves and Kratky transforms from 0.2-s exposures collected over 3.0 s. Linear Guinier transforms at initial (0.2 s) and final (3.0 s) exposures indicate absent aggregation.

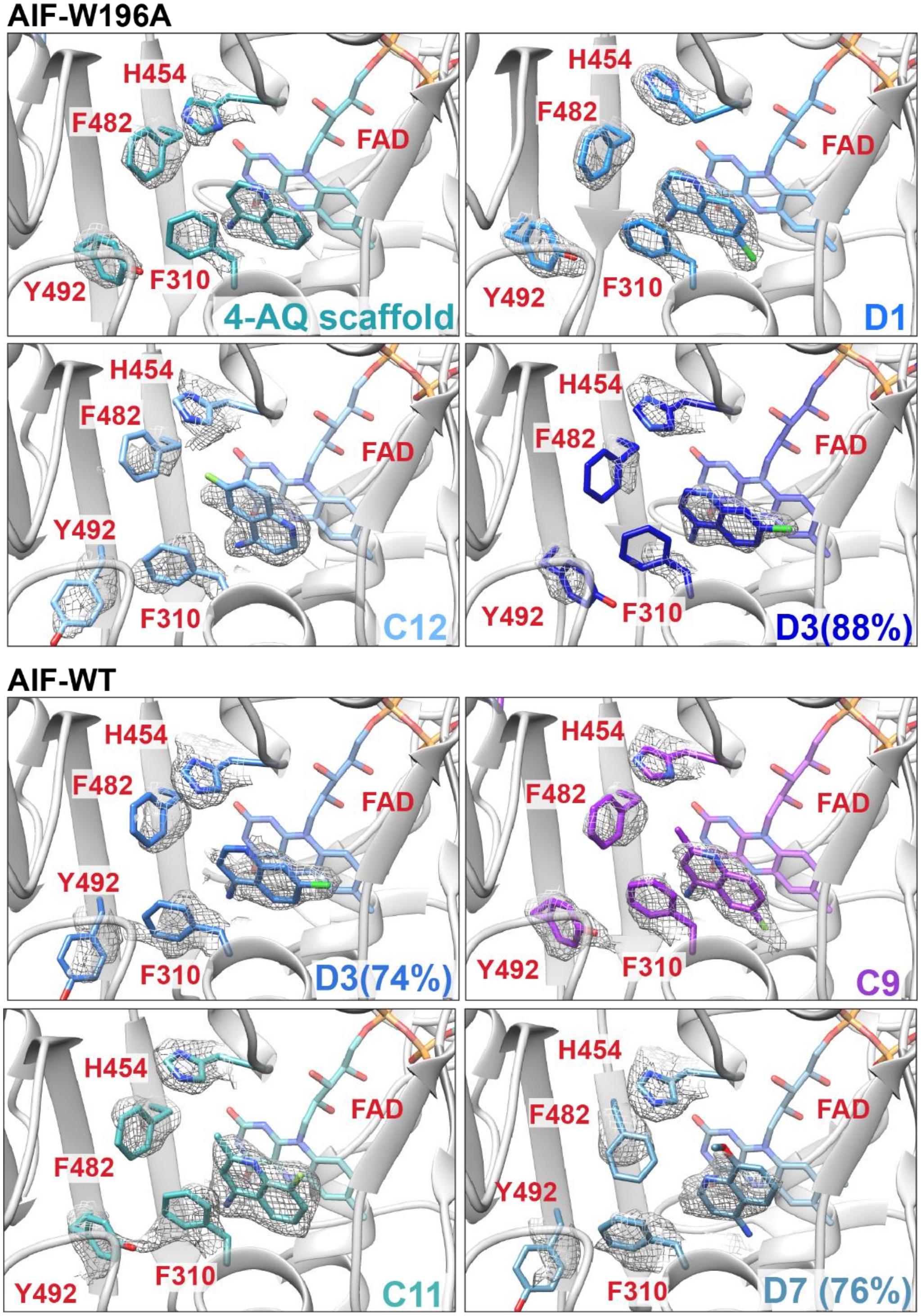

Supplementary Figure 10. AIF-WT and AIF-W196A active site SA-omit maps. Views of AIF's active site with simulated-annealing (SA) composite omit maps calculated with omission of ligands and residues H454, F482, F310, and Y492. Refined ligand occupancies less than 100% are indicated in parentheses. Maps are displayed at 1σ within 2.0 Å of selected ligands and residues for Monomer A with the exception of AIF-WT-C11, which displays Monomer B.

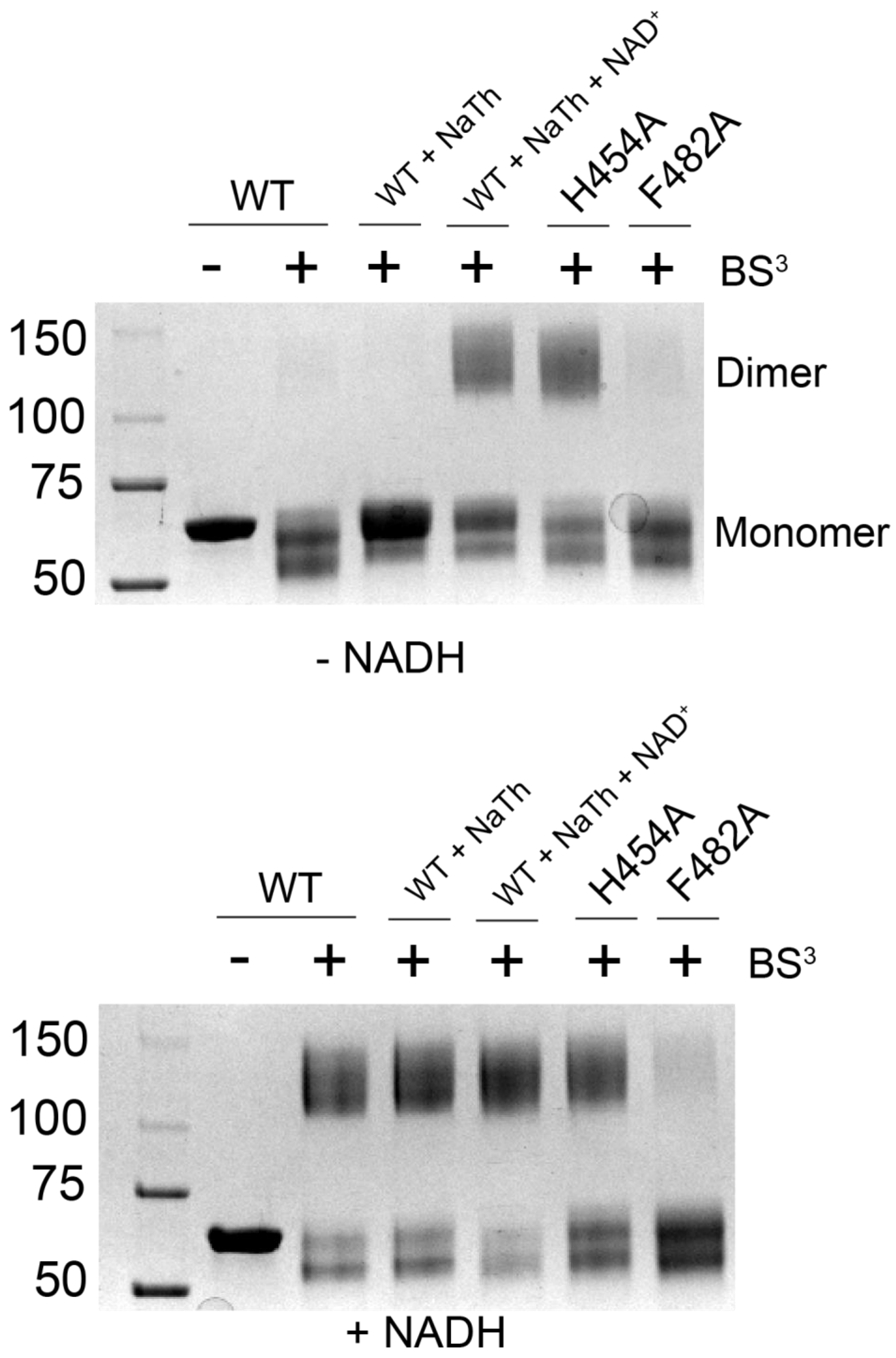

Supplementary Figure 11. Uncropped SDS-PAGE gels of wild-type and mutant AIF cross-linking reactions. Lanes containing AIF-WT and sodium dithionite (NaTh) are from a separate study.

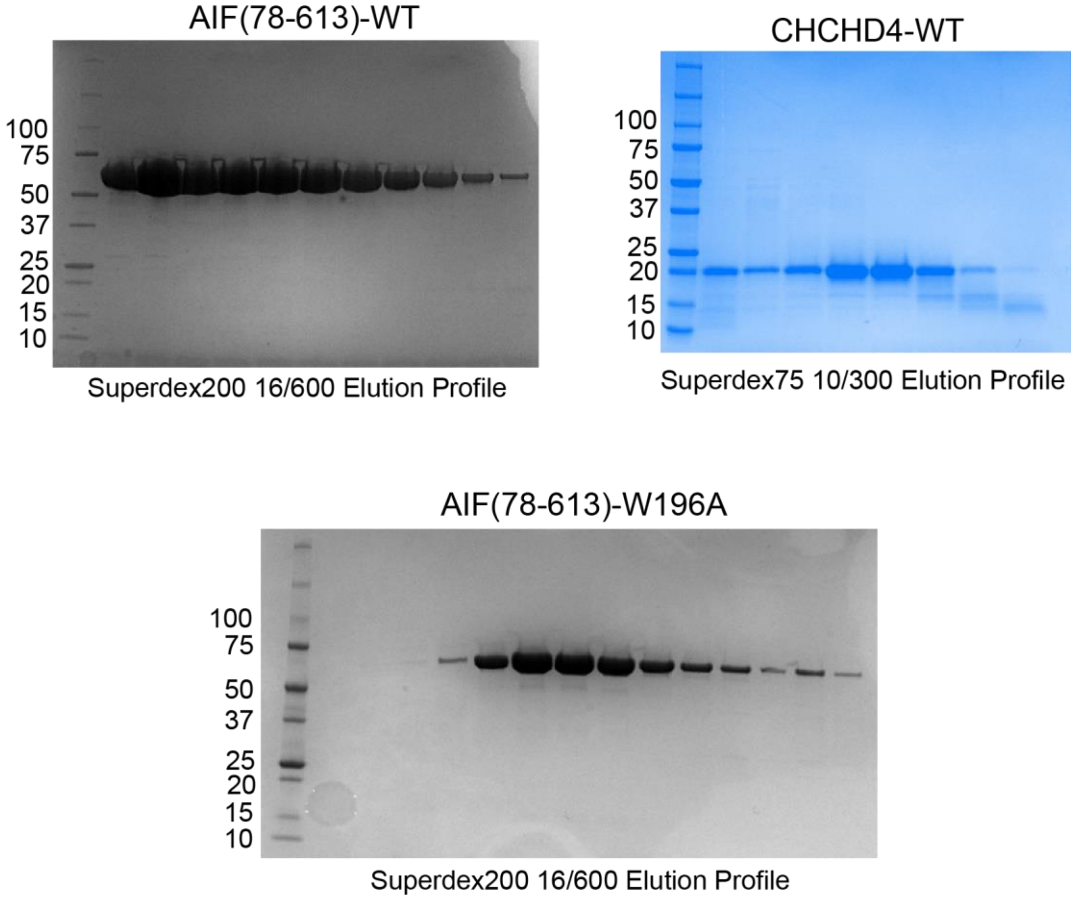

Supplementary Figure 12. SDS-PAGE analysis of AIF-WT, AIF-W196A, and CHCHD4 SEC profiles. AIF-WT and AIF-W196A run at their expected molecular weight of 60 kDa. The C-terminus of CHCHD4 is enriched in negatively charged amino acids causing it to run at a higher molecular weight relative to mass of 16 kDa. The displayed gels are representative of at least 3 independent protein purifications.

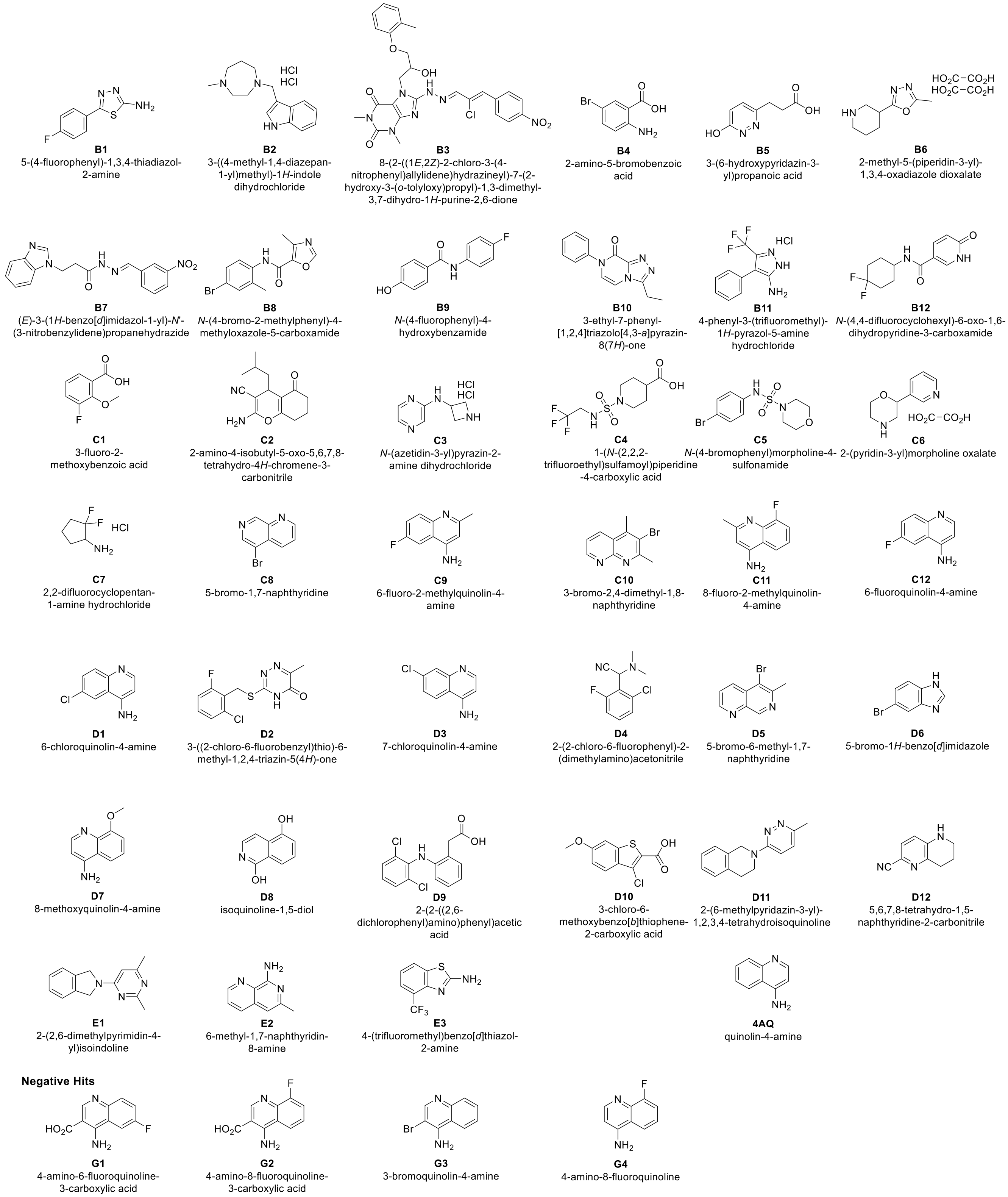

Supplementary Figure 13. Chemical 2D Diagrams of GL2500 Fragment Hits and 4-aminoquinoline scaffold. Overview of all chemical structures reported in the manuscript, including negative screening hits G1-G4.

AIF (78-613)-WT

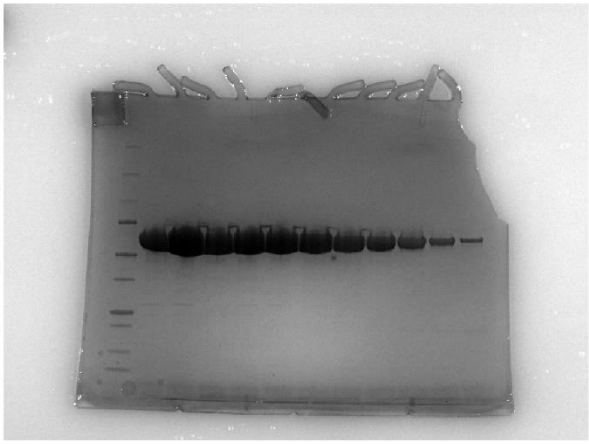

AIF (78-613)-W196A

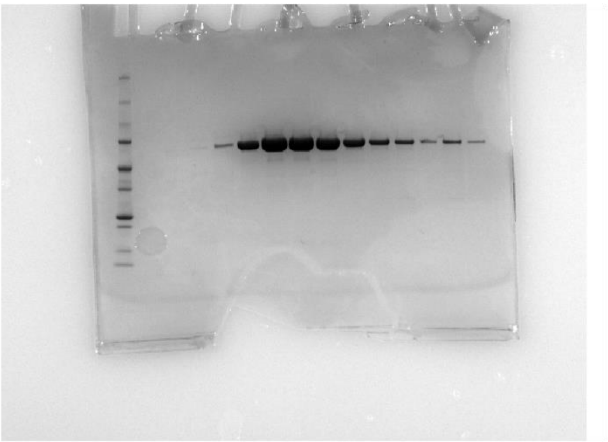

CHCHD4-WT

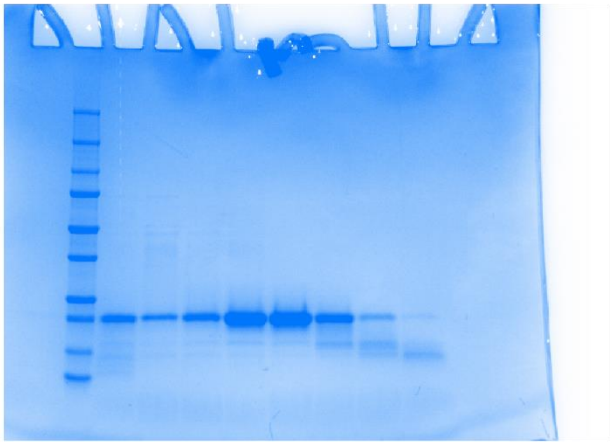

Supplementary Figure 14. Uncropped gel images from Supplementary Fig. 12.

Supplementary Table 1. Small Molecule Screening Data

Category	Parameter	Description
Assay	Type of assay	*In vitro* differential scanning fluorescence
	Target	Apoptosis-inducing factor, mitochondrial (AIFM1, Uniprot O95831)
	Primary measurement	Fluorescent detection of SYPRO Orange signal
	Key reagents	SYPRO Orange protein gel stain (5000X) ThermoFisher Scientific (Life Technologies), NADH (Sigma-Aldrich)
	Assay protocol	See Methods section - *High-throughput DSF Screening*
	Additional comments	Mashalidis, *et al* (doi:10.1038/nprot.2013.130) provides additional information for performing HT-DSF screening assays.
Library	Library size	2500 compounds
	Library composition	The GL2500 library is distributed among the following fragment libraries offered by Life Chemicals: brominated (19%), fluorinated (19%), protein–protein interaction disruptors (PPI) (15%), Fsp3-enriched (fragments containing sp3 hybridized carbon units) (19%), and Superior (fragments designed for solubility, low toxicity and cell permeability) (27%). Fragments were chosen for chemical diversity, absence of PAINS liabilities, and predicted favorable physicochemical properties.
	Source	Life Chemicals
	Additional comments	The library was purchased pre-dispensed into 96-deep-well blocks at 10 mg/mL (15-30 mM) in deuterated DMSO and stored at -30°C. Further details on library composition and its applications can be found in Moiani, *et al* (doi: 0.1016/bs.mie.2021.09.003).
Screen	Format	384-well PCR plates (Thermo Fisher)
	Concentration(s) tested	0.75-1.5 mM (0.5 mg/mL), 5% DMSO
	Plate controls	Each PCR plate contained 4 AIF controls repeated in triplicate – buffer, 5% DMSO, 83 µM NADH, 83 µM NADH + 5% DMSO
	Reagent/ compound dispensing system	Beckman Biomek FX liquid-handling system
	Detection instrument and software	Applied Biosystems QuantStudio 6 Flex Real-Time PCR instrument
	Assay validation/QC	Standard deviation of DMSO controls, CV across plates (see also Figure S1).

	Correction factors	N/A
	Normalization	N/A
	Additional comments	Optimization and production HT-DSF screens were performed at the former Center for Molecular Structure and Function (CMSF) core at M.D. Anderson Cancer Center
Post-HTS analysis	Hit criteria	Hits were selected as…
		$\lvert \Delta T_m \rvert = \lvert T_m(\text{compound}) - T_m(\text{AIF-DMSO-control}) \rvert$
		$\lvert \Delta T_m \rvert > 1.5 \times SD(\text{AIF-DMSO-control}, N=216)$
	Hit rate	1%, $\Delta T_m > 1.5 \times SD$ | 2.4%, $\Delta T_m < 1.5 \times SD$
	Additional assay(s)	Differential scanning fluorescence, microscale thermophoresis, small-angle X-ray scattering
	Confirmation of hit purity and structure	The following top-ranking hits were re-purchased from Life Chemicals for follow-up analysis: F2156-0068 (C12), F2156-0057 (D1), F9995-2431 (D3).
		The following hits were verified by X-ray crystallography in complex with AIF: F2156-0068 (C12), F2156-0057 (D1), F9995-2431 (D3), F2156-0070 (C9), F2156-0047 (C11), F2183-0014 (D7).
	Additional comments	

Supplementary Table 2. AIF HT-DSF GL2500 Fragment Hits. Screening and verification T_m shifts and MST amplitudes for the top 39 fragment-binders from the GL2500 HT-DSF screen. Fragments in bold exhibited binding in at least one verification assay. Significance thresholds of three standard deviations relative to AIF-DMSO are 1.7°C (HT-DSF), 0.7°C (DSF), and 960.5 response units (MST).

Compound	Group	Reference ID	HT-DSF ΔT_m(°C)	DSF ΔT_m(°C)	MST Amplitude
**F0345-3713**	**Fluorinated**	**B1**	**3.0**	**-0.5**	**966.3**
F6541-5066	Superior	B2	2.9	0.3	955.7
**F0823-0057**	**PPI**	**B3**	**2.8**	**-8.4**	**952.0**
F1980-0013	Brominated	B4	2.5	-0.1	955.6
**F1967-1331**	**PPI**	**B5**	**2.5**	**1.3**	**1003.1**
F2184-0232	Superior	B6	2.4	-0.1	951.1
**F1092-1243**	**PPI**	**B7**	**2.3**	**0.3**	**975.8**
**F0664-0094**	**Brominated**	**B8**	**2.2**	**1.4**	**952.7**
F3098-2193	Fluorinated	B9	2.1	-0.1	957.7
F3222-2571	Superior	B10	2.0	0.2	953.9
**F2130-0094**	**Fluorinated**	**B11**	**2.0**	**-0.3**	**963.5**
F6559-1405	Fluorinated	B12	1.9	0.3	952.3
**F0001-2710**	**Fluorinated**	**C1**	**1.9**	**0.8**	**954.5**
**F0894-0163**	**Fsp3**	**C2**	**1.8**	**0.7**	**953.5**
**F2167-2742**	**Superior**	**C3**	**1.7**	**0.7**	**970.6**
**F1906-0093**	**PPI**	**C4**	**1.7**	**2.1**	**952.7**
F3083-0166	Brominated	C5	1.7	-0.2	955.3
**F2135-1123**	**Superior**	**C6**	**1.7**	**0.8**	**957.2**
**F8888-6524**	**Fluorinated**	**C7**	**1.7**	**1.2**	**950.6**
**F1957-0058**	**Brominated**	**C8**	**-9.4**	**-9.9**	**955.1**
**F2156-0070**	**Fluorinated**	**C9**	**-5.6**	**-4.8**	**966.6**
**F1957-0061**	**Brominated**	**C10**	**-5.6**	**-6.5**	**954.5**
**F2156-0047**	**Fluorinated**	**C11**	**-5.0**	**-3.5**	**1036.9**
**F2156-0068**	**Fluorinated**	**C12**	**-4.6**	**-4.2**	**1022.3**
**F2156-0057**	**Superior**	**D1**	**-4.1**	**-3.3**	**958.9**
**F1894-0242**	**Fluorinated**	**D2**	**-3.8**	**-1.9**	**954.4**
**F9995-2431**	**Superior**	**D3**	**-3.7**	**-3.6**	**967.0**
**F2189-0161**	**Fluorinated**	**D4**	**-3.2**	**-2.1**	**951.1**
**F1957-0186**	**Brominated**	**D5**	**-2.9**	**-1.3**	**953.2**
**F2163-0004**	**Brominated**	**D6**	**-2.5**	**-2.0**	**959.9**
**F2183-0014**	**Superior**	**D7**	**-2.5**	**-3.2**	**978.3**
**F2135-0800**	**Superior**	**D8**	**-2.5**	**-2.7**	**974.6**
**F0722-0745**	**PPI**	**D9**	**-2.4**	**-1.7**	**961.0**
**F0818-0021**	**PPI**	**D10**	**-2.4**	**-0.7**	**965.7**
**F6541-1463**	**Superior**	**D11**	**-2.4**	**-1.5**	**969.9**
**F1957-0100**	**Superior**	**D12**	**-2.4**	**-2.9**	**986.0**
**F6543-8631**	**Superior**	**E1**	**-2.3**	**-1.3**	**952.0**
**F1957-0093**	**Superior**	**E2**	**-2.2**	**-2.2**	**991.5**
**F2169-0129**	**Fluorinated**	**E3**	**-2.2**	**-0.8**	**965.1**

Supplementary Table 3. SAXS Collection and Analysis Parameters

Sample Details	AIF-WT	AIF-W196A
Organism	*H. sapiens*	*H. sapiens*
Source	*E. coli* expressed (Brosey *et al.*, 2016[1])	*E. coli* expressed (Brosey *et al.*, 2016[1])
UniProt ID (residues in construct)	O95831 (78-613)	O95831 (78-613)
Extinction coefficient (FAD) (A_{450}, M^{-1} cm^{-1})	13,400[2]	13,400[2]
$\bar{v}$ from chemical composition (cm^3 g^{-1})	0.739	0.739
Particle contrast from sequence and solvent components, $\Delta\bar{\rho}$ ($\rho_{protein}$ − $\rho_{solvent}$; 10^{10} cm^{-2})	2.82 (12.23 – 9.46)	2.82 (12.23 – 9.46)
Mr from chemical composition (Da)	60,237	59,335
Solvent (buffer for subtraction taken from preparatory SEC flowthrough prior to elution of protein)	25 mM HEPES, pH 7.5, 150 mM NaCl	25 mM HEPES, pH 7.5, 150 mM NaCl
HT-SAXS samples concentrations	4 mg/mL	4 mg/mL
HT-SAXS sample volume	30 µL	30 µL

**SAXS Data-Collection Parameters**	
Instrument/data processing	Advanced Light Source (ALS) SIBYLS SAXS beamline (12.3.1) with Dectris Pilatus3 2M Detector[3]
Wavelength (Å)	1.27
Beam size (µm)	Converging beam, 500x2000 at sample, 100x100 at detector
Camera length (m)	1.5
q measurement range ($Å^{-1}$)	0.01–0.59
Absolute scaling method	On a detector scale
Normalization	To transmitted intensity by beam-stop counter
Monitoring for radiation damage	Data and R_g frame-by-frame comparison
Exposure time	Sequential 0.2-0.3 s exposures for 10 s.
Sample configuration	Samples were loaded by a multichannel Tecan Evo 100 liquid-handling robot into needle tips containing mica windows. Effective sample path length 1.5 mm.
Sample temperature (°C)	10
**SAXS Analysis Software**	
SAXS data reduction	Advanced Light Source (ALS) SIBYLS SAXS beamline (12.3.1) with Dectris Pilatus3 2M Detector[3]
Extinction coefficient estimate	*ProtParam*[4]
Calculation of $\bar{v}$ and $\Delta\bar{\rho}$	MULCh v 1.1.1[5] (https://smb-research.smb.usyd.edu.au/NCVWeb/index.jsp)
First-order analyses, I(0), R_g	ScÅtter (https://bl1231.als.lbl.gov/scatter/), Primus/qt ATSAS 3.0.1[6]
Graph visual display	Microsoft Excel, GraphPad Prism 9.0
**SIMPLE SCATTERING ID**	XS97QA1S (AIF Screening Panel at 0.3 s [oxidized] and 2.1 s [reduced])

Supplementary Table 3. SAXS Collection and Analysis Parameters (cont.)

Structural Parameters	AIF-WT (0.3 s)	AIF-WT (2.1 s)	AIF-WT (3.0 s)
Guinier Analysis			
I(0)	20.80 (0.19)	23.54 (0.23)	26.95 (0.28)
R_g (Å)	29.42 (0.39)	32.19 (0.44)	33.39 (0.48)
q_{min} (Å^{-1})	0.0137	0.0137	0.0137
qR_g max (q_{min} = 0.0137 Å^{-1})	1.29	1.30	1.29
Coefficient of correlation, R^2	1.00	1.00	0.96
Mass from Porod invariant (Da)[7]	45,582	51,347	52,669
Real-space Analysis			
I(0)	20.69 (0.69)	22.58 (0.72)	26.23 (1.42)
R_g (Å)	30.09 (0.85)	32.25 (0.95)	33.98 (1.47)
D_{max} (Å)	109	113	126
q range (Å^{-1})	0.0137 - 0.2338	0.0137 - 0.2338	0.0137 - 0.2338
χ^2	0.38	0.33	0.33
Porod volume, V_p (Å^3)	101,436	116.936	122,452
Porod coefficient, P_x	3.98 (0.15)	3.96 (0.13)	4.04 (0.11)

Structural Parameters	AIF-WT/ NADH (0.3 s)	AIF-WT / NADH (2.1 s)	AIF-WT / NADH (3.0 s)
Guinier Analysis			
I(0)	45.94 (0.49)	45.11 (0.49)	48.08 (0.53)
R_g (Å)	42.14 (0.59)	42.27 (0.62)	41.69 (0.61)
q_{min} (Å^{-1})	0.0125	0.0125	0.0125
qR_g max (q_{min} = 0.0125 Å^{-1})	1.30	1.28	1.29
Coefficient of correlation, R^2	1.00	1.00	0.99
Mass from Porod invariant[7]	100,900	99,497	103,410
Real-space Analysis			
I(0)	45.85 (0.99)	45.53 (0.82)	44.84 (1.05)
R_g (Å)	44.70 (2.00)	45.47 (1.62)	42.77 (2.43)
D_{max} (Å)	181	187	175
q range (Å^{-1})	0.0137 - 0.2338	0.0137 - 0.2338	0.0137 - 0.2338
χ^2	0.21	0.24	0.92
Porod volume, V_p (Å^3)	314,858	304,031	309,876
Porod coefficient, P_x	2.70 (0.03)	2.84 (0.03)	2.71 (0.05)

Structural Parameters	AIF-W196A (0.3 s)	AIF-W196A (2.1 s)	AIF-W196A (3.0 s)
Guinier Analysis			
I(0)	24.76 (0.22)	30.03 (0.31)	31.18 (0.35)
R_g (Å)	33.40 (0.40)	37.35 (0.49)	38.13 (0.54)
q_{min} (Å^{-1})	0.0101	0.0101	0.1010
qR_g max (q_{min} = 0.0101 Å^{-1})	1.30	1.30	1.29
Coefficient of correlation, R^2	1.00	1.00	1.00
Mass from Porod invariant[7]	71,332	90,153	95,054
Real-space Analysis			
I(0)	24.41 (0.87)	29.18 (0.58)	31.53 (0.71)
R_g (Å)	34.15 (0.95)	37.41 (0.85)	39.20 (1.18)
D_{max} (Å)	124	138	140
q range (Å^{-1})	0.0101 - 0.2142	0.0101 - 0.2142	0.0101 - 0.2142
χ^2	0.16	0.56	0.31
Porod volume, V_p (Å^3)	143,938	178,046	216,953
Porod coefficient, P_x	3.94 (0.11)	3.99 (0.08)	3.23 (0.03)

Supplementary Table 4. Wild-type and W196A AIF affinities for mitochondrial partner CHCHD4 stimulated by native NADH or CX1 chemotype ligands. Standard errors of fitting are included in parentheses. Binding constants for wild-type AIF with the CX1 chemotype are approximate as saturation was not reached.

	Dissociation Constants for CHCHD4 (µM)	
	**AIF-WT**	**AIF-W196A**
**DMSO**	ND	4.29 (0.30)
**NADH**	0.47 (0.02)	0.70 (0.04)
**C12**	46 (12)	1.54 (0.07)
**D1**	13 (1.2)	0.99 (0.11)
**D3**	12 (1.1)	0.99 (0.06)

Supplementary Table 5. AIF-GL2500 Summary of Crystallographic Structures.

Target	Chemotype	Ligand	Resolution (Å)	R_{work} / R_{free} (%)	Refined Ligand Occupancy Monomer A	Refined Ligand Occupancy Monomer B
**AIF-W196A**	1	C12	2.38	18.0 / 21.2	100%	Unmodelled
	1	D1	2.58	19.4 / 24.2	88%	77%
	1	D3	2.51	20.4 / 23.1	100%	100%
	1	4AQ	2.65	21.3 / 24.4	100%	100%
**AIF-WT**	1	D3	2.25	20.7 / 23.7	74%	77%
	2	C9	2.40	19.7 / 21.4	100%	100%
	2	C11	2.30	18.9 / 22.0	80%	100%
	2	D7	2.25	21.3 / 23.7	76%	76%

Supplementary Table 6. X-ray data collection and refinement statistics for AIF-W196A-GL2500 complexes

	AIF-W196A-C12	AIF-W196A-D1	AIF-W196A-D3	AIF-W196A-4AQ
**Data collection**				
Space group	$P2_12_12_1$	$P2_12_12_1$	$P2_12_12_1$	$P2_12_12_1$
Cell dimensions				
a, *b*, *c* (Å)	90.79, 114.33, 120.07	88.3, 110.97, 114.77	90.79, 114.77, 120.91	91.06, 114.97.122.03
α, β, γ (°)	90, 90, 90	90, 90, 90	90, 90, 90	90, 90, 90
Resolution (Å)	29.25-2.39 (2.47-2.39)	29.33-2.58 (2.67-2.58)	29.36-2.51 (2.6-2.51)	29.49-2.65 (2.75- 2.65)
R_{merge}	0.102 (0.712)	0.105 (0.77)	0.0839 (0.731)	0.128 (0.905)
$I / \sigma I$	16.1 (3.3)	14.4 (2.9)	18.7 (3.0)	15.4 (2.5)
Completeness (%)	99.3 (93.4)	99.7 (98.9)	99.5 (96.7)	99.9 (100.0)
Redundancy	10.7 (10.5)	9.9 (9.8)	10.4 (10.5)	11.0 (11.1)
**Refinement**				
Resolution (Å)	2.39	2.58	2.51	2.65
No. reflections	50130 (4649)	36094 (3543)	43661 (4182)	37870 (3728)
R_{work} / R_{free}	18.0% / 21.2%	19.4% / 24.2%	20.4% / 23.1%	21.3% / 24.3%
No. atoms	7006	6666	6802	6627
Protein	6523	6425	6488	6428
Ligand/ion	127	134	134	147
Water	356	107	180	52
B-factors	47.46	59.98	62.67	55.34
Protein	47.71	60.35	63.02	55.55
Ligand/ion	36.85	49.35	56.77	49.43
Water	46.55	50.6	54.57	46.13
R.m.s. deviations				
Bond lengths (Å)	0.004	0.005	0.004	0.004
Bond angles (°)	0.59	0.62	0.59	0.59

*Values in parentheses are for highest-resolution shell.

Supplementary Table 7. X-ray data collection and refinement statistics for AIF-WT-GL2500 complexes

	AIF-WT-D3	AIF-WT-C9	AIF-WT-C11	AIF-WT-D7
**Data collection**				
Space group	$P2_12_12_1$	$P2_12_12_1$	$P2_12_12_1$	$P2_12_12_1$
Cell dimensions				
a, *b*, *c* (Å)	91.32, 115.22, 122.48	91.47, 115.32, 123.97	91.39, 115.02, 124.12	91.10, 115.05, 122.17
α, β, γ (°)	90, 90, 90	90, 90, 90	90, 90, 90	90, 90, 90
Resolution (Å)	73.21-2.25 (2.33-2.25)	84.44-2.4 (2.49-2.4)	57.51-2.3 (2.38-2.3)	73.03-2.25 (2.33-2.25)
R_{merge}	0.118 (1.26)	0.0887 (0.712)	0.0756 (0.656)	0.0750 (0.874)
$I / \sigma I$	12.89 (1.69)	19.85 (3.25)	21.63 (3.58)	17.75 (2.20)
Completeness (%)	99.9 (99.9)	99.8 (99.8)	99.9 (99.8)	99.5 (99.0)
Redundancy	8.9 (9.1)	10.4 (9.8)	13.2 (13.4)	11.2 (10.9)
**Refinement**				
Resolution (Å)	2.25	2.4	2.3	2.25
No. reflections	61911 (6074)	51865 (5113)	58686 (5762)	61308 (6011)
R_{work} / R_{free}	20.7% / 23.5%	19.9% / 21.4%	19.0% / 22.0%	21.3% / 23.7%
No. atoms	6977	6820	6960	6828
Protein	6526	6487	6554	6508
Ligand/ion	168	161	161	176
Water	283	172	245	144
B-factors	53.75	54.44	53.71	64.61
Protein	54.02	54.76	54.04	64.99
Ligand/ion	48.28	48.18	47.01	58.8
Water	50.67	48.15	49.43	54.42
R.m.s. deviations				
Bond lengths (Å)	0.005	0.004	0.005	0.004
Bond angles (°)	0.64	0.59	0.66	0.55

*Values in parentheses are for highest-resolution shell.

Supplementary References

1 Brosey, C. A. *et al.* Defining NADH-Driven Allostery Regulating Apoptosis-Inducing Factor. *Structure* **24**, 2067-2079, doi:10.1016/j.str.2016.09.012 (2016).

2 Churbanova, I. Y. & Sevrioukova, I. F. Redox-dependent changes in molecular properties of mitochondrial apoptosis-inducing factor. *J Biol Chem* **283**, 5622-5631, doi:10.1074/jbc.M709147200 (2008).

3 Classen, S. *et al.* Implementation and performance of SIBYLS: a dual endstation small-angle X-ray scattering and macromolecular crystallography beamline at the Advanced Light Source. *Journal of Applied Crystallography* **46**, 1-13, doi:10.1107/S0021889812048698 (2013).

4 Gasteiger, E. *et al.* in *The Proteomics Protocols Handbook* (ed John M. Walker) 571-607 (Humana Press, 2005).

5 Whitten, A. E., Cai, S. & Trewhella, J. MULCh: modules for the analysis of small-angle neutron contrast variation data from biomolecular assemblies. *Journal of Applied Crystallography* **41**, 222-226, doi:doi:10.1107/S0021889807055136 (2008).

6 Franke, D. *et al.* ATSAS 2.8: a comprehensive data analysis suite for small-angle scattering from macromolecular solutions. *J Appl Crystallogr* **50**, 1212-1225, doi:10.1107/S1600576717007786 (2017).

7 Rambo, R. P. & Tainer, J. A. Accurate assessment of mass, models and resolution by small-angle scattering. *Nature* **496**, 477-481, doi:10.1038/nature12070 (2013).
