# Supplementary material for: Chemical screening by time-resolved X-ray scattering to discover allosteric probes
Source: Nat Chem Biol. Author manuscript; Available in PMC 2024 Sep 1. (PMC11358040; doi:10.1038/s41589-024-01609-1)
Supplement: Reporting Summary [file NIHMS2003799-supplement-Reporting_Summary.pdf]

nature portfolio

Corresponding author(s): John A. Tainer

Last updated by author(s): Mar 8, 2024

Reporting Summary

Nature Portfolio wishes to improve the reproducibility of the work that we publish. This form provides structure for consistency and transparency in reporting. For further information on Nature Portfolio policies, see our Editorial Policies and the Editorial Policy Checklist.

Statistics

For all statistical analyses, confirm that the following items are present in the figure legend, table legend, main text, or Methods section.

n/a	Confirmed	
☐	☒	The exact sample size (*n*) for each experimental group/condition, given as a discrete number and unit of measurement
☐	☒	A statement on whether measurements were taken from distinct samples or whether the same sample was measured repeatedly
☒	☐	The statistical test(s) used AND whether they are one- or two-sided<br>*Only common tests should be described solely by name; describe more complex techniques in the Methods section.*
☒	☐	A description of all covariates tested
☒	☐	A description of any assumptions or corrections, such as tests of normality and adjustment for multiple comparisons
☐	☒	A full description of the statistical parameters including central tendency (e.g. means) or other basic estimates (e.g. regression coefficient) AND variation (e.g. standard deviation) or associated estimates of uncertainty (e.g. confidence intervals)
☒	☐	For null hypothesis testing, the test statistic (e.g. *F*, *t*, *r*) with confidence intervals, effect sizes, degrees of freedom and *P* value noted<br>*Give P values as exact values whenever suitable.*
☒	☐	For Bayesian analysis, information on the choice of priors and Markov chain Monte Carlo settings
☒	☐	For hierarchical and complex designs, identification of the appropriate level for tests and full reporting of outcomes
☒	☐	Estimates of effect sizes (e.g. Cohen's *d*, Pearson's *r*), indicating how they were calculated

Our web collection on statistics for biologists contains articles on many of the points above.

Software and code

Policy information about availability of computer code

Data collection	DSF melt curves were collected with commercial software provided with ThermoFisher's QuantStudio Flex 6 and the BioRad CFX Connect Real-Time PCR System.<br><br>MST binding curves were collected and processed with PR.ThermControl 2.1.6 and PR.Stability Analysis 1.0.2 software provided with NanoTemper's Monolith NT.115 system.<br><br>UV-visible absorbance spectra were acquired with commercial software provided with Agilent Technologies' Cary 60 UV-vis spectrophotometer- Scan application version 5.1.0.1016 [Firmware version 6.4.0.141. Hardware version 2.00].<br><br>X-ray diffraction and small-angle X-ray scattering data were collected using standard software packages provided by the associated synchrotron beamline (cited in Methods): Blu-Ice v September 2019, July 2020 (ALS BL8.3.1); Life Science Data Collection (LSDC) v Nov. 2020, Oct. 2021 (NSLS-II FMX, 17-ID-2); Blue-Ice/DCS v May 2017 (ALS SYBILS 12.3.1).
Data analysis	Melting temperatures for DSF melt curves were calculated with GraphPad Prism 9.0 (HT-DSF) or BioRad's CFX Maestro software (v 1.0) (verification DSF).<br><br>MST binding curves were normalized with NanoTemper's MO.Affinity Analysis software and fit to a one-site binding model with GraphPad Prism 9.0<br><br>SAXS data were analyzed were analyzed with Primus 3.0.1 and ScÅtter 3.0, and SAXS parameters derived from these programs were further analyzed and prepared for visualization in Microsoft Excel (Version 2303) and GraphPahd Prism 9.0. SAXS similarity matrix maps and volatility-

of-ration (VR) values were calculated using the SAXS similarity web application on the SIBYLS website (https://sibyls.als.lbl.gov/saxs-similarity/), then clustered using the application's single-linkage agglomerative hierarchical clustering (AHC) algorithm. VR values were independently clustered using a k-means approach with the NbClust function in R (v.4.3.1). Rate constants for time-evolved volatility-of-ratio values were calculated with a single decaying exponential in GraphPahd Prism 9.0.

X-ray diffraction data were processed with fast_dp (FMX) or XDS (v Mar 2019) and CCP4 (v 7.0) (BL 8.3.1) Molecular replacement and refinement utilized the Phaser and refinement modules of Phenix 1.18.2. Model building and correction were carried out with Coot (v. 0.8.9). Molecular visualization and analysis of X-ray structures were done with UCSF Chimera (v. 1.14).

For manuscripts utilizing custom algorithms or software that are central to the research but not yet described in published literature, software must be made available to editors and reviewers. We strongly encourage code deposition in a community repository (e.g. GitHub). See the Nature Portfolio guidelines for submitting code & software for further information.

Data

Policy information about availability of data

All manuscripts must include a data availability statement. This statement should provide the following information, where applicable:

- Accession codes, unique identifiers, or web links for publicly available datasets
- A description of any restrictions on data availability
- For clinical datasets or third party data, please ensure that the statement adheres to our policy

Structural coordinates used for crystallographic molecular replacement calculations and AIF images (Fig. 1b) were accessed from the World Wide Protein Data Bank (https://www.rcsb.org/) from entries 4BV6 (AIF-WT, monomer), 5KVH (AIF-W196A), and 4BUR (AIF-WT-NADH, dimer). Structural coordinates and crystallographic structure factors of AIF-aminoquinoline complexes have been deposited with the World Wide Protein Data Bank (https://www.rcsb.org/) as follows: AIF-W196A-C12 (PDB: 8D3E), AIF-W196A-D1 (PDB: 8D3G), AIF-W196A-D3 (PDB: 8D3H), AIF-W196A-4AQ (PDB: 8D3I), AIF-WT-D3 (PDB: 8D3N), AIF-WT-C9 (PDB: 8D3J), AIF-WT-C11 (PDB: 8D3K), AIF-WT-D7 (PDB: 8D3O). AIF-ligand SAXS datasets from 0.3 s (oxidized) and 2.1 s (reduced) exposures have been deposited with the SIMPLE SCATTERING repository (https://simplescattering.com/) under the code XS97QA1S. The authors declare that the data supporting the findings of this study are available within the paper and its associated source data files and Supplementary Information files. Should any raw data files be needed in another format they are available from the corresponding author upon reasonable request.

Human research participants

Policy information about studies involving human research participants and Sex and Gender in Research.

Reporting on sex and gender	The manuscript does not include human subject research.
Population characteristics	The manuscript does not include human subject research.
Recruitment	The manuscript does not include human subject research.
Ethics oversight	The manuscript does not include human subject research.

Note that full information on the approval of the study protocol must also be provided in the manuscript.

Field-specific reporting

Please select the one below that is the best fit for your research. If you are not sure, read the appropriate sections before making your selection.

☒ Life sciences ☐ Behavioural & social sciences ☐ Ecological, evolutionary & environmental sciences

For a reference copy of the document with all sections, see nature.com/documents/nr-reporting-summary-flat.pdf

Life sciences study design

All studies must disclose on these points even when the disclosure is negative.

Sample size	Samples sizes were not calculated, since this study design did not involve population-wide analysis or allocation into experimental groups. Statistical analyses reported in this study include standard curve-fitting, defining HT-DSF/DSF/MST screening cut-offs based upon averaged AIF-DMSO controls (N>3), fitting errors associated with SAXS parameters (Supplementary Table 3), and X-ray diffraction processing and refinement statistics (Supplementary Tables 6-7), all as described in the Methods.
Data exclusions	HT-DSF screening data points were excluded for melting curves exhibiting fluorescence interference or signs of sample aggregation using pre-established criteria as described in the Methods. SAXS data were excluded for scattering curves exhibiting signs of poor background subtraction, aggregation, or sample bubbles as described in the Methods.
Replication	HT-DSF hits were verified or eliminated by secondary DSF or MST screening. The time-dependent stimulation of AIF dimerization by the three CX1 SAXS compounds was verified by 3 independent SAXS experiments.

Randomization	Randomization is not relevant to this study as our research did not involve allocation into experimental groups.
Blinding	Blinding is not relevant to this study, since group allocation was not used. The nature of our analysis is such that results are uninfluenced by researcher blinding.

Reporting for specific materials, systems and methods

We require information from authors about some types of materials, experimental systems and methods used in many studies. Here, indicate whether each material, system or method listed is relevant to your study. If you are not sure if a list item applies to your research, read the appropriate section before selecting a response.

Materials & experimental systems

n/a	Involved in the study
☒	☐ Antibodies
☒	☐ Eukaryotic cell lines
☒	☐ Palaeontology and archaeology
☒	☐ Animals and other organisms
☒	☐ Clinical data
☒	☐ Dual use research of concern

Methods

n/a	Involved in the study
☒	☐ ChIP-seq
☒	☐ Flow cytometry
☒	☐ MRI-based neuroimaging
