# Supplementary figures and images for: Chemical screening by time-resolved X-ray scattering to discover allosteric probes
Source: Nat Chem Biol. Author manuscript; Available in PMC 2024 Sep 1. (PMC11358040; doi:10.1038/s41589-024-01609-1)

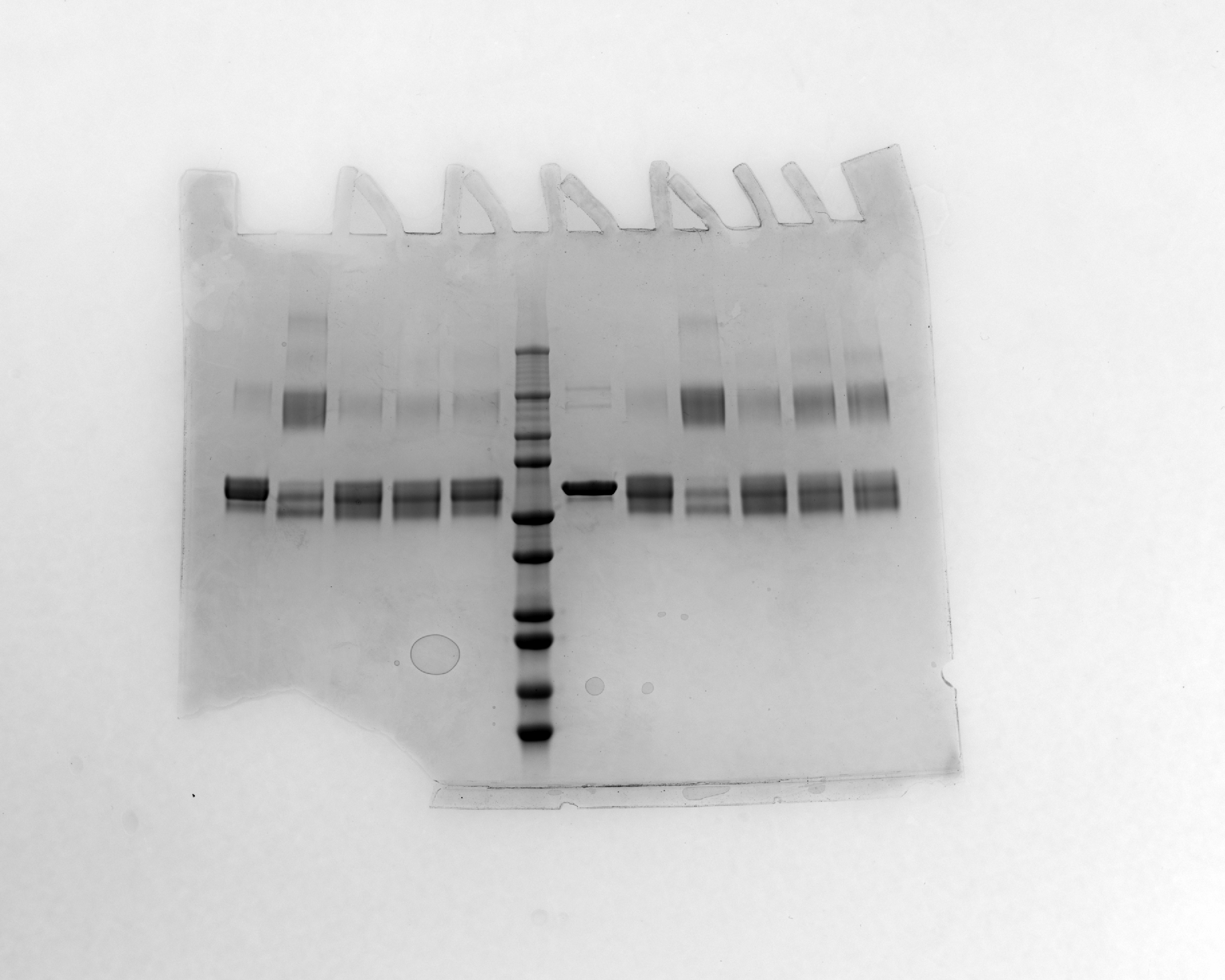

Supplement: Source Data Fig. 4 [file NIHMS2003799-supplement-Source_Data_Fig__4.zip › Unmodified_Gels_Fig4a.jpg]

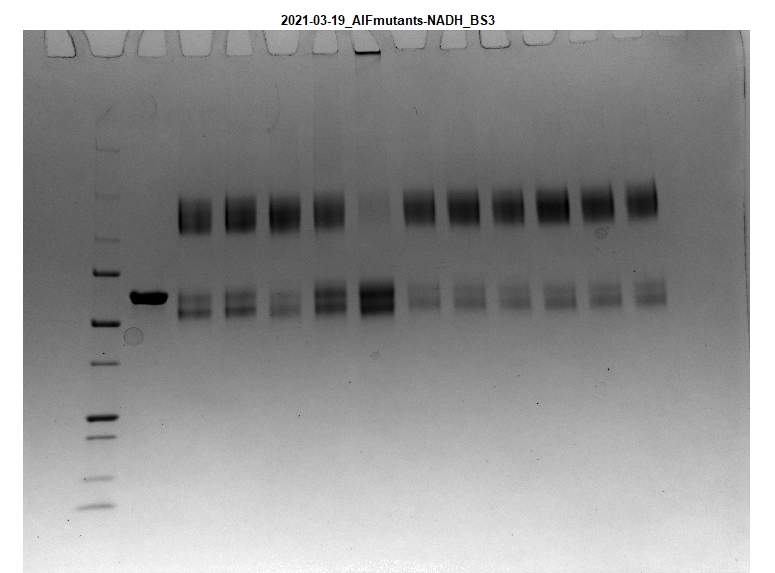

Supplement: Source Data Fig. 5 [file NIHMS2003799-supplement-Source_Data_Fig__5.zip › Unmodified_Gel_Fig5d_NADH.jpg]

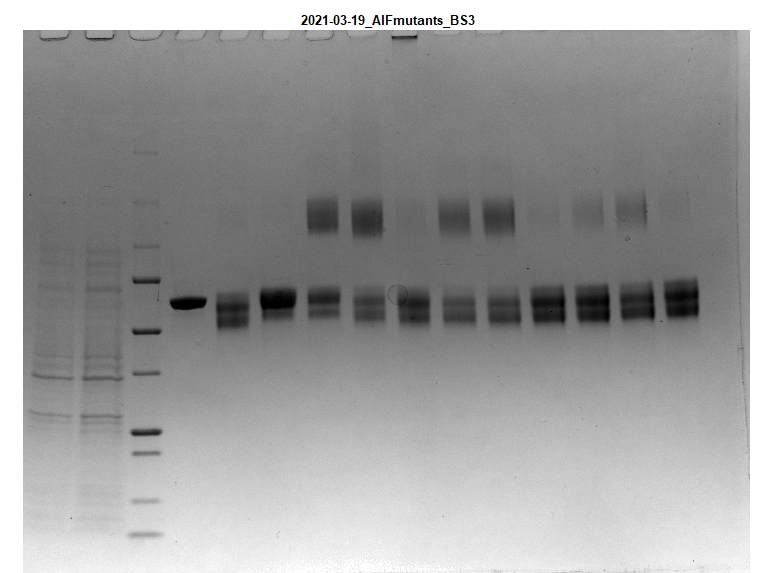

Supplement: Source Data Fig. 5 [file NIHMS2003799-supplement-Source_Data_Fig__5.zip › Unmodified_Gel_Fig5d_NoNADH.jpg]
